# Fluorescent tagging of endogenous Heme oxygenase-1 in human induced pluripotent stem cells for high content imaging of oxidative stress in various differentiated lineages

**DOI:** 10.1007/s00204-021-03127-8

**Published:** 2021-09-04

**Authors:** Kirsten E. Snijders, Anita Fehér, Zsuzsanna Táncos, István Bock, Annamária Téglási, Linda van den Berk, Marije Niemeijer, Peter Bouwman, Sylvia E. Le Dévédec, Martijn J. Moné, Rob Van Rossom, Manoj Kumar, Anja Wilmes, Paul Jennings, Catherine M. Verfaillie, Julianna Kobolák, Bas ter Braak, András Dinnyés, Bob van de Water

**Affiliations:** 1 Division of Drug Discovery and Safety, Leiden Academic Centre for Drug Research, Leiden University, Einsteinweg 55, 2333 CC Leiden, The Netherlands; 2 BioTalentum Ltd., 2100 Gödöllő, Hungary; 3 Department of Physiology and Animal Health, Institute of Physiology and Animal Health, Hungarian University of Agriculture and Life Sciences, 2100 Gödöllő, Hungary; 4 Department of Development and Regeneration, Stem Cell Institute, KU Leuven, Leuven, Belgium; 5 Division of Molecular and Computational Toxicology, Amsterdam Institute for Molecules, Medicines and Systems, Amsterdam, The Netherlands

**Keywords:** Oxidative stress, Reporter cells, Induced pluripotent stem cells, In vitro toxicology, Endogenous gene tagging, High content imaging

## Abstract

Tagging of endogenous stress response genes can provide valuable in vitro models for chemical safety assessment. Here, we present the generation and application of a fluorescent human induced pluripotent stem cell (hiPSC) reporter line for Heme oxygenase-1 (HMOX1), which is considered a sensitive and reliable biomarker for the oxidative stress response. CRISPR/Cas9 technology was used to insert an enhanced green fluorescent protein (eGFP) at the C-terminal end of the endogenous *HMOX1* gene. Individual clones were selected and extensively characterized to confirm precise editing and retained stem cell properties. Bardoxolone-methyl (CDDO-Me) induced oxidative stress caused similarly increased expression of both the wild-type and eGFP-tagged HMOX1 at the mRNA and protein level. Fluorescently tagged hiPSC-derived proximal tubule-like, hepatocyte-like, cardiomyocyte-like and neuron-like progenies were treated with CDDO-Me (5.62–1000 nM) or diethyl maleate (5.62–1000 µM) for 24 h and 72 h. Multi-lineage oxidative stress responses were assessed through transcriptomics analysis, and HMOX1-eGFP reporter expression was carefully monitored using live-cell confocal imaging. We found that eGFP intensity increased in a dose-dependent manner with dynamics varying amongst lineages and stressors. Point of departure modelling further captured the specific lineage sensitivities towards oxidative stress. We anticipate that the newly developed HMOX1 hiPSC reporter will become a valuable tool in understanding and quantifying critical target organ cell-specific oxidative stress responses induced by (newly developed) chemical entities.

**Supplementary Information:**

The online version contains supplementary material available at 10.1007/s00204-021-03127-8.

## Introduction

Newly generated pharmaceuticals and chemicals need to be assessed for their potential toxic effects in humans. Over the last decades, a variety of reporter systems have been developed as alternatives of animal models for the prediction of chemical-induced toxicities (Scrivens and Bhogal 2007), including systems based on transformed cell lines and primary cell cultures (Collet et al. 2019; Michael 2017; Sonneveld et al. 2005). In vitro reporter assays not only provide relevant toxicological information, but also allow high-throughput screening of potential toxicants (Hiemstra et al. 2019; Wink et al. 2017). However, transformed cell lines may be defective in their response to certain toxicants, since they often have mutations in toxicologically relevant pathways such as P53 (Jennings 2015). While primary cells do not suffer this issue, they have a limited proliferation capacity, supply is often limited and they can have variability and quality issues (Jennings 2015; Levy et al. 2015).

More recently, it has been suggested that induced pluripotent stem cells (iPSCs) may offer a good alternative to traditional toxicology assessments (Goldring et al. 2017; Liu et al. 2017; Suter-Dick et al. 2015). iPSCs are characterized by their capacity of long-term self-renewal and differentiation potential to all lineages, representing an unlimited source of organ-specific cells (Takahashi et al. 2007; Yu et al. 2007). They provide a unique advantage for testing organ-specific sensitivities from cells carrying the same genetic background, offering great potential to refine organ-specific toxicology models.

Well defined genes involved in stress response pathways (e.g. oxidative stress, DNA damage and endoplasmic reticulum stress) are good candidates as toxicology biomarkers. The development of reporter cell lines as in vitro models by endogenous tagging of such marker genes can represent valuable screening platforms for toxicology studies (Hiemstra et al. 2019; Wink et al. 2014, 2018). Oxidative stress is defined as an imbalance between the production and elimination of reactive oxygen species (ROS). Increased level of free radicals is toxic, damaging all components and macromolecules of the cells. The Kelch-like ECH-associated protein 1/nuclear factor erythroid 2-related factor 2 (KEAP1/NRF2) signalling pathway is known to be the key controller of the redox homeostasis by activating the transcription of cytoprotective genes involved in antioxidant stress response (Kensler et al. 2007; McMahon et al. 2003; Zhang 2006). These downstream effectors such as intracellular redox-balancing proteins like HMOX1 and NAD(P)H Quinone Dehydrogenase 1 (NQO1) have a crucial role in the redox-state maintenance and cellular defence mechanisms (Baird and Dinkova-Kostova 2011). In this study, we focused on HMOX1, which is a tail-anchored (TA) protein localized mainly in the endoplasmic reticulum (ER) membrane (Dunn et al. 2014; Lee et al. 2016; Maines 1997). The major function of this protein is the degradation of the pro-oxidative heme, that is released from heme proteins upon oxidative stress, which is then transformed to biliverdin and immediately converted to bilirubin, a strong antioxidant and free-radical scavenger molecule (Dulak and Jozkowicz 2014; Kikuchi et al. 2005). HMOX1 is a stress-inducible protein activated by the NRF2 and AP-1 transcription factors (Paine et al. 2010), upregulated under pro-oxidant conditions at both the mRNA and protein level (Baird and Dinkova-Kostova 2011; Wilmes et al. 2011). HMOX1 is, therefore, widely accepted as a sensitive and fairly ubiquitous marker of oxidative stress which, therefore, could be an excellent candidate for reporter development in hiPSCs enabling high-throughput quantification of HMOX1 upregulation during chemical-induced stress (Attucks et al. 2014; Choi and Alam 1996; Deng et al. 2015; Ryter et al. 2006).

Previously established reporter systems generated by conventional random integration of promoter-driven reporter genes were not favourable due to a number of limitations (Liu 2013). Location of the insertion site can influence the reporter expression leading to inadequate epigenetic modifications and altered regulation (Kwaks and Otte 2006; Yáñez and Porter 2002), and multiple integrations of the transgene may result in overexpression artefacts or inadequate expression patterns (Doyon et al. 2011). Furthermore, the length of the promoter that drives the expression of a particular gene is often unknown or not well defined, and the important regulatory regions can extend to hundreds of kilobases. Whilst some of these limitations can be overcome using Bacterial Artificial Chromosomes (BACs), (Poser et al. 2008), this system has very poor efficiency in hiPSCs due to the large construct size. We, therefore, incorporated the reporter gene, enhanced green fluorescent protein (eGFP), into the native *HMOX1* genomic locus to retain the characteristic expression profile of the endogenous protein in the cell. CRISPR/Cas9 (Clustered Regularly Interspersed Short Palindromic Repeat Associated protein 9) technology represents a widely used and powerful way for precise genome editing (Cong et al. 2013; Doudna and Charpentier 2014; Jinek et al. 2012; Mali et al. 2013). The system is based on the generation of a site-specific DNA double-strand break by Cas9 nuclease mediated DNA-cleavage under the guidance of a single guide RNA (sgRNA/gRNA). The incorporation of exogenous DNA sequences into the target locus and the generation of knock-ins can be achieved when the DNA damage is repaired through the high-fidelity homology-directed repair pathway (HDR) (Jasin and Rothstein 2013). The frequency of HDR is cell type-dependent, and in hiPSCs extremely low (He et al. 2016; Yang et al. 2013). Due to the low HDR-efficiency in hiPSCs, the insertion of relatively long DNA like the coding sequence of fluorescent reporter genes is still challenging (Roberts et al. 2017). Nevertheless, endogenous protein tagging, where the reporter is under the physiological regulatory control of the native protein, will undoubtedly provide the most specific stress response readout (Dambournet et al. 2014; Ratz et al. 2015).

Here, we report the endogenous tagging of HMOX1 in hiPSCs by CRISPR/Cas9 genome editing and present the complete characterization and functional validation of the generated cell line, which can be coupled with cell lineage-specific differentiations in combination with high content imaging (HCI) platforms to serve as a precious multi-organ oxidative stress reporter test system in toxicology studies.

## Materials and methods

### Chemicals

Chemicals were purchased from Merck KGaA (Darmstadt, Germany), and cell culture reagents and culture plates were purchased from Thermo Fisher Scientific (Waltham, MA, USA), unless specified otherwise.

### hiPSC culture

The hiPSC line SBAD2 clone 1, derived from Normal Adult Human Dermal Fibroblasts (NHDF-Ad) cells (Lonza, 51 years old Caucasian male dermal fibroblast cells, Cat. No: CC-2511) were reprogrammed with non-integrative Sendai virus transduction, obtained during the course of the IMI-funded StemBANCC project (stembancc.org) (Morrison et al. 2015). Cells were cultured at 37 °C in a humidified atmosphere containing 5% CO_2_ in a feeder-free system on tissue culture dishes and plates coated with Matrigel (BD Biosciences). Cells were grown in mTeSR-1 medium (StemCell Technologies Inc.) and passaged every 5–7 days using EDTA (0.02%, Versene, Lonza). For imaging and compound exposures, hiPSCs were dissociated into single cells using 1X TrypLE Select, then 62,500 cells/cm^2^ were seeded into Matrigel-coated 96-well microplates (Greiner Bio-One) and the culture medium was supplemented with 1X RevitaCell for 24 h. 48 h post-seeding hiPSCs were ready for exposure. hiPSCs underwent routine mycoplasma screening and karyotyping.

### Gene targeting

Once SBAD2 hiPSC cultures reached 70–80% confluency, they were incubated with Accutase (Sigma–Aldrich) at 37 °C for 9 min to prepare single-cell suspension for genome editing; then 8 × 10^5^ cells were nucleofected with CRISPR/Cas9 RNP complex (4.5 µg) and donor vector (2 µg) using Human Stem Cell Nucleofector Kit 1 (Lonza) and program B-016 in AMAXA Nucleofector^™^ 2b Device (Lonza). After nucleofection, the cells were seeded in a 6-well plate and 1X RevitaCell Supplement was added into the mTeSR-1 culture medium to increase cell recovery. Puromycin selection started 2 days later by supplementing the media with 0.8 µg/ml puromycin (Thermo Fisher Scientific) on the first day, then increased to 1 µg/ml for another 4 days. Following selection, puromycin-resistant colonies were isolated and transferred into organ dishes. After separate propagation, cells were harvested for cryopreservation and for DNA analysis.

### Cassette removal

1 × 10^6^ *HMOX1*-targeted hiPSCs (clone H7-03) were nucleofected with 2 µg Excision Only PiggyBac™ Transposase Expression Vector (SBI) then plated onto 10 cm dishes for colony picking. 10 days after nucleofection, individual colonies were isolated and transferred into organ dishes for separate propagation. DNA analysis and puromycin-sensitivity testing of the cells were performed after three passages.

### Western blot

Cells were lysed with RIPA Lysis and Extraction Buffer supplemented with Halt™ Protease and Phosphatase Inhibitor Cocktail and Pierce™ Universal Nuclease for Cell Lysis (Thermo Fisher Scientific), then the samples were sonicated. The concentration of the isolated proteins was determined using BCA Protein Assay Kit (Pierce). 2 µg protein was separated on 12% SDS–polyacrylamide gels and transferred to PVDF membranes (Bio-Rad). Membranes were blocked with 5% low-fat milk in TBS-Tween, then incubated overnight with primary antibodies against HMOX1 (Cell Signaling Technology, CST#70081), eGFP (Cell Signaling Technology, CST#2956) and GAPDH (Sigma, G9545) at 4 °C; followed by the appropriate HRP-conjugated secondary antibody (Cell Signaling Technology, CST#7074) for 1 hour at room temperature. Signals were detected with SuperSignal^™^ West Dura Extended Duration Substrate using KODAK Gel Logic 1500 Imaging System (Bruker).

### Directed differentiation of hiPSCs

SBAD2 HMOX1-eGFP hiPSCs were differentiated using previously established growth factor based differentiation protocols to generate day 28 hepatocyte-like cells (Boon et al. 2020), day 21 cardiomyocyte-like cells (van den Berg et al. 2016), day 21 neuron-like cells (Chambers et al. 2009; Shi et al. 2012) and day 14 proximal tubule-like cells (Chandrasekaran et al. 2021). Detailed procedures are described in the supplementary materials.

### Compound exposure

SBAD2 HMOX1-eGFP reporter cells were exposed for 24 h or 72 h to ten concentrations of bardoxolone methyl (CDDO-Me, CAS# 218600-53-4), diethyl maleate (DEM, CAS# 141-05-9) and 0.2% DMSO vehicle control. CDDO-Me stock concentrations of 500 µM were prepared in DMSO (CAS# 67-68-5). DEM stocks of 2 mM were prepared freshly on the day in medium containing 0.4% DMSO. Concentrations ranging from 5.62 to 1000 nM (CDDO-Me) or 5.62 to 1000 µM (DEM) were prepared in 96-well deep-well plates through serial dilutions of quarter-log increments. 50 µL of exposure medium containing 2X the desired end concentration was added on top of the 50 µL culture medium in each well. One technical replicate was included per exposure condition and two for the controls. All exposures were completed in triplicate on cells originating from three independent differentiations.

### TempO-Seq transcriptomic analysis

To determine cell lineage specification, three technical replicates were collected from untreated controls for each of the three independent differentiations. Samples were also collected following 24 h CDDO-Me or DEM exposure, with two biological replicates per exposure condition. For collection, cells were washed with 200 µL 1X PBS (Sigma) and lysed with 50 µL 1X BNN lysis buffer (BioSpyder, Carlsbad, USA) for 15 min at room temperature. Lysates were frozen at − 80 °C and sent for TempO-Seq analysis (Yeakley et al. 2017) to Bioclavis (Glasgow, UK) of a targeted gene set consisting of the S1500 + gene list (Mav et al. 2018) supplemented with genes involved in cellular stress responses and differentiation markers (so-called EU-ToxRisk gene panel; Supplementary Table 5). A sequencing depth of 1.5 million reads per sample was used resulting in a minimal average read depth of ~ 500 reads per gene. Raw reads were aligned using the TempO-Seq R package by Bioclavis. Read counts were normalized using counts per million (CPM) and log_2_ transformed, followed by differential expression analysis using the DESeq2 R package (Love et al. 2014). Samples were excluded for further analysis when having a library size of lower than 100,000 counts, reducing the technical replicates to two for one biological replicate of one of the untreated control samples. Differentially expressed genes (DEG) were defined as having an adjusted *p* value lower than 0.05 based on a Wald test using the DESeq2 R package. Compound exposure samples were compared to lineage-specific DMSO 0.2% samples for DEG determination. For lineage marker assessment, log_2_ fold change (log_2_FC) was calculated for untreated controls of differentiated compared to undifferentiated hiPSC, for which the top 25 most upregulated and top 5 most downregulated DEGs were chosen. Gene functionality was categorized according to GeneCards.org. Within the EU-ToxRisk gene panel, target genes of oxidative stress response transcription factor NRF2 were identified as defined by DoRothEA v2 (Garcia-Alonso et al. 2019, 2018) using confidence A to C. Heatmaps were generated for data visualisation and rows were clustered using Euclidean distance similarity metric. R packages used for analysis are as previously described (ter Braak et al. 2021).

### High content confocal imaging

For the identification of nuclei during high content imaging, cells were incubated for 2 h prior to compound exposures with nucleic acid stain Hoechst 33342 (H1399, Thermo Fisher Scientific) using an end concentration of 0.1 µg/ml. To enable the detection of necrosis, 0.1 nM of propidium iodide (PI) was added to the compound exposure media. Live confocal microscopy was performed with a Nikon Eclipse Ti microscope at 5% CO_2_ and 37 °C using a 20X objective. Automated imaging acquired images at nine positions per well every 1 h over 24 h using NIS software (Nikon, Amsterdam, The Netherlands). Excitation by 408, 488 and 561 nm lasers resulted in emission detection of the Hoechst nuclear signal, cytoplasmic HMOX1-eGFP and PI, respectively. To avoid oversaturation of the induced eGFP signal caused by varying basal levels of HMOX1, laser settings were adjusted between lineages to ensure no eGFP signal was present at time point 0 h.

### Image quantification and normalization

Image quantification was done using CellProfiler 2.1 (Broad Institute RRID:SCR_007358) where segmentation pipelines (Wink et al. 2017) were adjusted to account for lineage-specific morphologies. Using an in-house R package (Wink et al. 2014), the quantified single-cell data were normalized as follows. Well positions containing less than 100 cells at time point 0 h were excluded, leaving three images or more for further analysis. For sparse cardiomyocyte-like cells this cell number threshold was not applied. For all lineages, nuclear counts at time point 0 h were subtracted from nuclear counts at all time points to represent the nuclear increase. To account for laser degradation between replicates, the mean eGFP intensity per imaged well was min–max normalized $\left( {zi\; = \;\left( {xi - \min \left( x \right)/\left( {\max \left( x \right) - \min \left( x \right)} \right)} \right)} \right)$, where per plate the maximum eGFP intensity at 24 h for CDDO-Me was used as maximum and eGFP intensity at time point 0 h as minimum, hereby removing any basal HMOX1 expression present before exposure. Due to a technical laser malfunction, no data was obtained for neuron-like cells replicate 3 for time points 12 until 19 h. The obtained data were fitted and missing data points were extrapolated using the B-spline function (Perperoglou et al. 2019). To obtain the PI positive fraction, cells that exhibited a PI positive signal in > 10% of the area of the nucleus were counted and divided by the total number of cells. Fraction of PI positive cells at 0 h were subtracted from all data points to normalize the data.

### Statistical analysis

Unless otherwise stated experiments were performed in triplicate and statistical differences were calculated based on the standard error of the mean. Rstudio version 1.1.456 (Boston, USA) and R 3.4.1 were used for data analysis and figure generation. R packages included dplyr (Wickham et al. 2018), ggplot2 (Wickham 2016) and pheatmap (Kolde 2019).

## Results

### Strategy for HMOX1-tagging and gene targeting

**Fig. 1:**
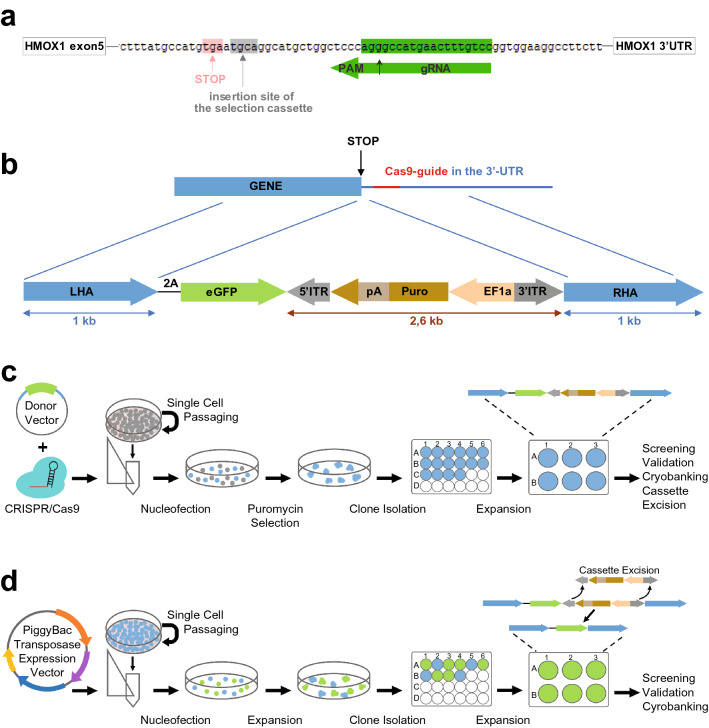
Generation of the SBAD2-HMOX1-eGFP fluorescent reporter hiPSC line. **a** gRNA sequence, selected for HMOX1-tagging, targeting the 3′-UTR of the gene. Stop codon of HMOX1, insertion site of the selection cassette and the CRISPR/Cas9 cleavage site are indicated by arrows. **b** The eGFP protein was inserted in frame to the C-terminal end of HMOX1 and separated by the 2A peptide as shown by the donor vector architecture. The selection cassette was placed into the 3′-UTR and contained a puromycin resistance gene driven by EF1α-promoter. **c** Pipeline for CRISPR/Cas9 knock-in of eGFP donor vector and subsequent clonal selection. **d** Strategy for removal of selection cassette using the Excision Only PiggyBac Transposase Expression Vector followed by clonal isolation and validation of the reporter line

To establish hiPSCs carrying a HMOX1-eGFP reporter, we performed CRISPR/Cas9-assisted eGFP-tagging with a ribosome-skipping 2A peptide between the biomarker’s C-terminus and the fluorophore, enabling reporter expression to be regulated by the endogenous *HMOX1* gene promoter. The applied donor vector contained a minimal selection cassette providing puromycin resistance to the edited cells and the cassette was flanked by PiggyBac-repeats allowing removal of the resistance gene later (Fig. 1). Three *HMOX1* targeting gRNAs were tested in vitro (Supplementary Table 1), and the gRNA closest to the end of the *HMOX1* gene was selected for CRISPR/Cas9 assisted knock-in of the donor vector. The TGCA sequence, indicated in Fig. 1a, was determined to be the most suitable place for cassette insertion and removal, due to its close proximity to the *HMOX1* stop codon and similarity to the PiggyBac footprint sequence (TTAA) causing minimal change in the original sequence.

Cas9 protein was precomplexed with the gRNA, after which the RNP-complexes and the donor vector were transfected into SBAD2 hiPSCs by AMAXA-nucleofection. Cells were left to recover for two days and subsequently underwent puromycin selection to eliminate the cells without successful vector-integration (Fig. 1c). After selection, drug-resistant colonies were isolated, propagated and analysed further.

### Screening and clone testing after gene targeting

At first, junction PCRs were performed to screen for clones with correct genomic integration in the targeted *HMOX1* locus. Genotyping PCRs were designed to generate two overlapping amplicons spanning the entire inserted sequence at the target site. As a result, five precisely edited clones were found among the 29 tested clones (Supplementary Fig. 1a). The junction PCR-positive clones were then further screened for the genome-integrated vector copy number using eGFP-specific TaqMan assay and three potential single-copy clones were identified (Supplementary Fig. 1b). Southern blot analysis demonstrated the correct targeting event and homogeneity of the H7-03 clone, whilst the other two single-copy candidates proved to be heterogeneous, originated from mixed colonies (Supplementary Fig. 2). Junction regions were checked by Sanger-sequencing to confirm accurate on-target editing of *HMOX1* and the untagged allele was also verified to identify potential indels introduced via NHEJ. The H7-03 clone was confirmed to be mutation free in the target region on both the eGFP-tagged and untagged *HMOX1* allele. Non-specific CRISPR/Cas9 activity was analysed for the most likely predicted off-target cleavage sites and the results showed perfect matches, intact sequences without any insertions/deletions at those sites (Supplementary Table 2).

### Excision of the selection cassette

To generate “scarless” reporter cells, we removed the EF1*α*-promoter-driven puromycin selection cassette from the H7-03 SBAD2 hiPSC clone. The cells were nucleofected with an excision-competent but integration-defective PiggyBac vector (Li et al. 2013) and after transient transposase expression individual colonies were isolated, propagated and analysed. Subclones that underwent successful cassette removal were identified by PCR-genotyping (Supplementary Fig. 3a), further verified through Southern blot analysis (Supplementary Fig. 2) and tested for loss of puromycin resistance. Based on the findings, the H703-17 subclone was selected for further characterization, hereafter referred to as SBAD2-HMOX1-eGFP reporter hiPSC line.

### Characterization of the SBAD2-HMOX1-eGFP reporter hiPSC line

The HMOX1-eGFP reporter line was subjected to detailed genetic characterization. Sanger-sequencing confirmed the correct DNA sequence of the eGFP-tagged *HMOX1* allele and the successful cassette removal by the transposase (Supplementary Fig. 3b). Southern blot analysis showed a consistent result with this (Supplementary Fig. 2). The SBAD2-HMOX1-eGFP reporter cells displayed a normal diploid 46, XY karyotype as shown by Giemsa-banding (Supplementary Fig. 3c).

In addition to the genetic characterization, an important consideration when manipulating hiPSCs is the maintenance of pluripotency. To confirm the multi-lineage differentiation ability of the reporter cells, embryoid bodies were formed and cultured for 14 days in differentiation medium. The differentiated progeny was characterized for the expression of the three germ layer markers, both at protein and RNA (scorecard^™^) level. After eGFP-tagging, SBAD2 hiPSCs expressed pluripotency markers and were able to differentiate into ecto-, endo- and mesodermal lineages (Supplementary Fig. 4a), hereby confirming retained stem cell properties. We found no significant difference in the marker expression profile between the unedited and reporter SBAD2 cells (Supplementary Fig. 4b, c).

**Fig. 2:**
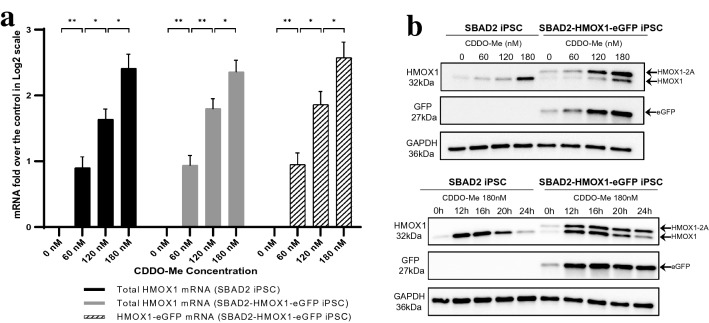
Effects of oxidative stress induction on the endogenous and eGFP-tagged HMOX1 expression in the SBAD2-HMOX1-eGFP reporter hiPSCs. **a** *HMOX1* mRNA expression in unedited SBAD2 and SBAD2-HMOX1-eGFP reporter iPSCs after 12 h CDDO-Me exposure, evaluated by RT-qPCR. The expression values are presented as mean ± SEM (*n *= 4) and calculated as a relative amount of the total *HMOX1* or *HMOX1-eGFP* mRNA versus the expression value of the untreated iPSCs, which was fixed to 1. One-way ANOVA was used to assess the statistical significance of the differences (*p < 0.05, **p < 0.01). **b** Western blot analysis of SBAD2 and SBAD2-HMOX1-eGFP hiPSCs after exposure to four CDDO-Me concentrations for 24 h (*n *= 3) or 180 nM CDDO-Me sampled at different timepoints (*n *= 3). HMOX1-2A/HMOX1: the 2A amino acid sequence remains at the C-terminus of HMOX1 resulting a slower-migrating form and allowing to distinguish between HMOX1 expressed from the tagged and wild-type alleles

To further characterize the HMOX1-eGFP hiPSC line and prove its functionality we tested the HMOX1 induction and eGFP reporter expression using bardoxolone methyl (CDDO-Me, CAS# 218600-53-4) as a strong NRF2/oxidative stress inducer. Unedited (’parental’ SBAD2) and HMOX1-eGFP reporter hiPSCs were exposed to increasing concentrations of CDDO-Me and analysed by RT-qPCR after 12 h exposure (Fig. 2a). Upon CDDO-Me treatment, total *HMOX1* mRNA levels increased significantly in both cell lines. Using specific primers for the eGFP-tagged *HMOX1* transcript, we found that gene expression from the tagged allele in the reporter hiPSCs was regulated similarly to that of the untagged endogenous *HMOX1* (Fig. 2a). Western blot analysis was performed after 24 h of CDDO-Me exposure and indicated a clear dose–response on protein level between the CDDO-Me concentrations and the HMOX1 and eGFP reporter expression (Fig. 2b, Supplementary Fig. 5). HMOX1 protein expressed from the eGFP-tagged allele can be distinguished from the endogenous wild-type protein due to its increased size caused by the 2A peptide on the tagged HMOX1. The Western blot analysis showed that HMOX1 protein expression from the wild-type allele was very similar and comparable in the unedited and reporter cell lines. We found, however, that expression of HMOX1 from the eGFP-tagged allele was induced to a higher level when compared to the wild-type allele expression, suggesting a higher stability for the 2A-tagged form of the protein. The reporter eGFP expression followed a similar trend as HMOX1, particularly resembling the induction of the tagged HMOX1 allele. Overall, the dose response to CDDO-Me treatment was clearly detectable in the expression of HMOX1 from both the wild-type and eGFP-tagged allele as well as in the expression of eGFP.

To investigate the time dynamics of the HMOX1 induction and eGFP expression upon oxidative stress, we treated the unedited and reporter hiPSCs with 180 nM CDDO-Me and analysed the samples by Western blot at different time points over a 24 h period (Fig. 2b, Supplementary Fig. 5). We found that the level of HMOX1 peaked after 12–16 h exposure, followed by a gradual decrease. In parallel, the eGFP expression reached saturation at the same time point and showed no further significant changes, remaining at that level until the end of the 24 h treatment, most likely due to a relatively higher eGFP protein-stability compared to HMOX1. Our results show that the SBAD2-HMOX1-eGFP reporter hiPSCs respond to oxidative stress in a robust and timely manner.

### Multi-lineage differentiation of SBAD2-HMOX1-eGFP

**Fig. 3:**
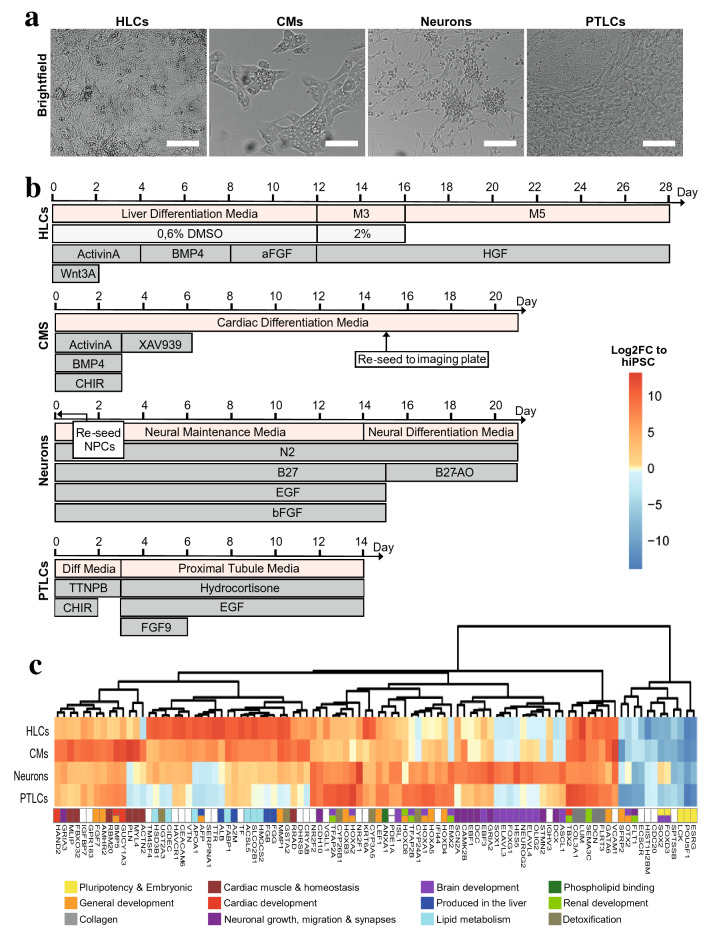
Multi-lineage differentiation of SBAD2-HMOX1-eGFP hiPSC reporter line. **a** Brightfield images depicting the morphology of SBAD2-HMOX1-eGFP hiPSCs after differentiation into hepatocyte-like cells (HLCs), cardiomyocyte-like cells (CMs), neuron-like cells (neurons) and proximal tubule-like cells (PTLCs). Scale bar is 100 µm. **b** Differentiation culture conditions and duration. HLCs were differentiated directly in 96-well plate for 28 days. CMs were differentiated for 15 days then reseeded into 96-well plates till day 21. Neural progenitor cells (NPCs) were plated onto 96-well plates and matured for a further 21 days. PTLCs underwent lineage specification for 14 days. **c** TempO-Seq analysis of lineage-specific markers after SBAD2-HMOX1-eGFP differentiation. Samples include untreated control conditions for HLCs, CMs, neurons and PTLCs. Per lineage, differentially expressed genes (DEGs) were selected that showed the 25 highest and 5 lowest log_2_ fold change (log_2_FC) compared to hiPSCs. DEGs were clustered according to the Euclidean distance metric. For all samples *n *= 3. Genes are color coded according to functionality

Having confirmed the reporter functionality, we next set out to assess the full potential of hiPSCs for toxicology assessments by representing known target organs of toxicity. For this purpose, SBAD2-HMOX1-eGFP cells were differentiated into hepatocyte-like cells (HLCs), cardiomyocyte-like cells (CMs), neuron-like cells and proximal tubule-like cells (PTLCs) (Fig. 3a, b). Using TempO-Seq transcriptomic analysis, we captured distinct branches containing differentially expressed genes (DEGs) specific to one lineage which indicated the successful differentiation of the SBAD2-HMOX1-eGFP cells towards the desired lineages (Fig. 3c).

*TF, TTR, ALB* and *CYP3A5* expression were found in HLCs, whose differential expression profiles included DEGs involved in lipid metabolism, detoxification or encoding for proteins produced in the liver. *ACTN2*, *MYL4, MLIP* and *PLN* displayed the most lineage-specific expression for the CMs (Giacomelli et al. 2017), together with DEGs involved in cardiac development, cardiac muscle and cardiac homeostasis. *HES5*, *FOXG1*, *ELAVL3* and *SOX1* expression were limited to neuron-like cells (Bansod et al. 2017; Ogawa et al. 2018; Vasconcelos and Castro 2014), alongside DEGs linked to brain development, neuronal migration, action potential and synapses. PTLCs expressed renal developmental markers *TBX2*, *EMX2*, *TFAP2A and TFAP2B* as well as Homeobox (*HOX*) genes, regulating developmental segment orientation (Bhatlekar et al. 2018)*.* Renal exclusive expression was seen for *SEMA3C, ANXA1* and *CYP24A1* (Vitamin D receptor), which is expressed preferentially in human renal proximal tubule epithelial cells in vivo (Banu et al. 2006; Reidy and Tufro 2011; Sadashiv et al. 2019; Sheikh and Solito 2018). hiPSC differentiation was coupled with a down regulation of pluripotency markers *ESRG, POU5F1, LCK, FOXD3, SOX2* and *OTX2* (Kim et al. 2014a; Yang et al. 2014) with the exception of neuron-like cells which partially retained neuroectodermal *FOXD3*, *SOX2* and *OTX2* expression (Zhou et al. 2014). Overall, fluorophore tagging did not affect the hiPSC’s differentiation potential and the expected expression profiles were obtained for all lineages tested (Fig. 3a–c).

### TempO-Seq analysis of multi-lineage oxidative stress induction

**Fig. 4:**
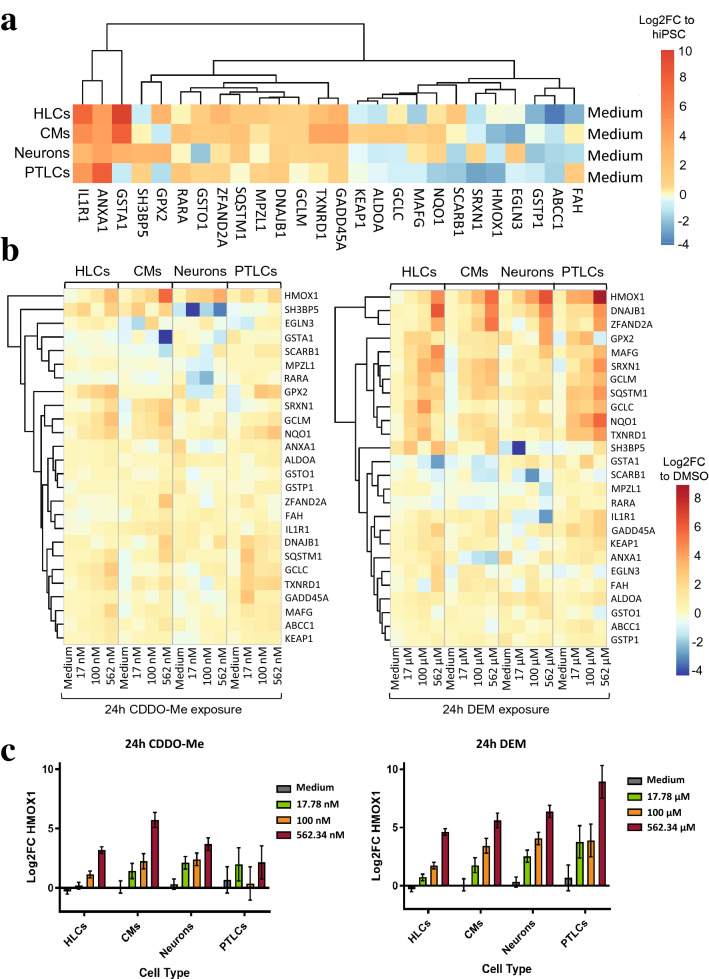
TempO-Seq analysis of oxidative stress response genes in hiPSCs-derived lineages. **a** TempO-Seq expression of NRF2 target genes selected based on the Dorothea downstream target selection tool, confidence A–C. Untreated control samples of four hiPSC-derived lineages displayed as log_2_ fold change (log_2_FC) compared to undifferentiated hiPSCs where *n *= 3. Lineages include hepatocyte-like cells (HLCs), cardiomyocyte-like cells (CMs), neuron-like cells (neurons) and proximal tubule-like cells (PTLCs). **b** TempO-Seq results of differentiated SBAD2-HMOX1-eGFP lineages after 24 h CDDO-Me and DEM exposure where *n *= 2. Displayed as log_2_FC compared to lineage-specific DMSO 0.2% sample. Differentially expressed genes represent NRF2 targets, clustered according to the Euclidean distance metric. **c** TempO-Seq results of *HMOX1* expression in differentiated SBAD2-HMOX1-eGFP lineages after 24 h CDDO-Me and DEM exposure. Log_2_FC of *HMOX1* was normalized per lineage to DMSO 0.2% vehicle control. Error bars indicate SEM and *n *= 2

To uncover how different cell types are primed towards the oxidative stress response we expanded our transcriptomic analysis to include several NRF2 target genes as defined by DoRothEA v2 (Garcia-Alonso et al. 2018, 2019) and calculated log_2_FCs in relation to undifferentiated hiPSCs (Fig. 4a). Overall, PTLCs displayed low basal NRF2 target gene expression, whereas this was most abundant for CMs, displaying the increased presence of *MAFG, GCLC, ALDOA, KEAP1* and *TXNRD1* in particular. Neuron-like cells were the only lineage to highly express *SRXN1* and *EGLN3*, the latter a known regulator of neuronal apoptosis (Lee et al. 2005). In line with important role of the liver in xenobiotic detoxification, HLCs expressed high basal levels of *GSTA1*. Hereby potentially indicating a cell-specific preconditioning for effective detoxification by GSH conjugation and regulation of the oxidative stress response.

To evaluate lineage-specific NRF2 regulation during compound exposure, we exposed the differentiated reporter cells to two different oxidative stress inducers, CDDO-Me or DEM, for 24 h. For each lineage, log_2_FC was calculated in relation to the DMSO solvent control (Fig. 4b). Both stressors activated the oxidative stress response in a dose-dependent manner with DEM overall inducing higher log_2_FCs and upregulating more NRF2 target genes than CDDO-Me in all lineages. HLCs uniquely displayed a drop in log_2_FC expression for *GCLM, GCLC, NQO1* and *TXNRD1* at the highest DEM concentration, outlining differences in activation mechanisms between lineages. Basal NRF2 target gene expression levels were indicative for lineage sensitivity to DEM exposure, with PTLCs displayed the most abundant gene activation coupled with low basal levels and CMs exhibiting the least activation and highest basal levels (Fig. 4a, b).

Overall, *HMOX1* was found to be the most sensitive NRF2 target, displaying dose-dependent induction independent of lineage or stressor, hereby confirming its efficacy as a biomarker for oxidative stress (Fig. 4a–c). To asses if this response was limited to hiPSC-derived lineages the HLC response was compared to primary human hepatocytes and HepG2 cells (Supplementary Fig. 6). The *HMOX1* response consistently appeared to be the most conserved out of all the NRF2 targets and clear dose-dependent induction of *HMOX1* was seen in all three liver test systems. Therefore, we have high confidence in the hiPSC HMOX1 reporter model for in vitro evaluation of liabilities for oxidative stress induction.

### Application of SBAD2-HMOX1-eGFP hiPSCs for high content imaging

**Fig. 5:**
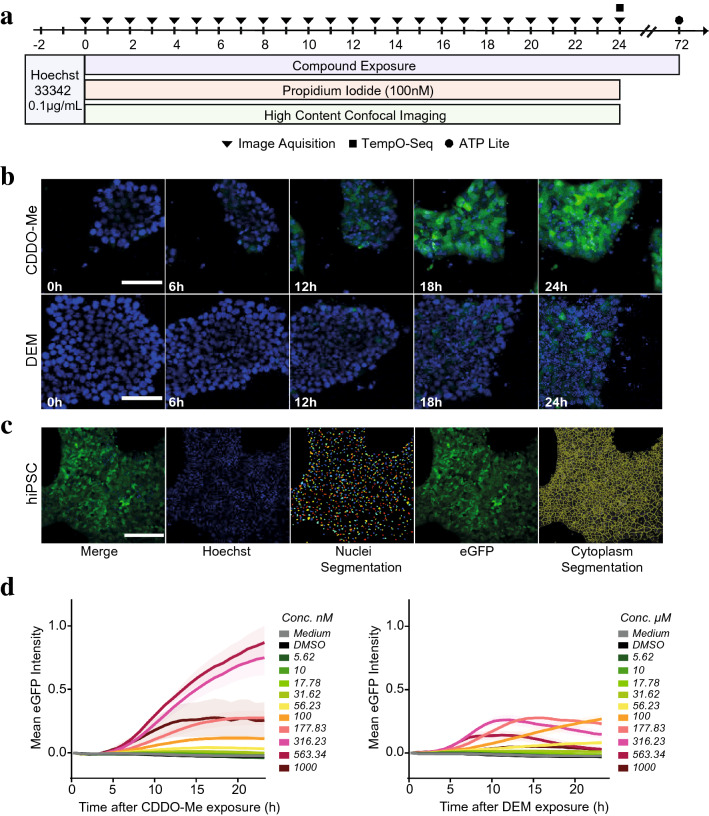
Functional validation of SBAD2-HMOX1-eGFP hiPSC reporter using high content confocal imaging. **a** Experimental set up of compound exposure and sample collection. Nuclei were stained for 2 h with 0.1 µg/ml Hoechst 33342 after which CDDO-Me or DEM compound media and cell death marker propidium iodide (PI) was added to the cells. HMOX1-eGFP reporter activity was assessed over 24 h using confocal imaging, at 24 h TempO-Seq samples were collected and at 72 h viability was assessed with ATP Lite. **b** Confocal images of SBAD2-HMOX1-eGFP reporter activity after exposure with 316.23 nM CDDO-Me and 100 µM DEM over 24 h. Hoechst stained nuclei visualised in blue and cytoplasmic eGFP visualised in green. Scale bar is 100 µm. **c** Example of object segmentation for image quantification on SBAD2-HMOX1-eGFP hiPSCs exposed to CDDO-Me for 24 h. HMOX1-eGFP channel was used for cytoplasmic segmentation (green), whilst nuclear segmentation was performed on the Hoechst channel (blue). Scale bar is 200 µm. **d** Quantified single-cell expression of eGFP during 24 h exposure to ten concentrations of CDDO-Me and DEM, alongside medium and DMSO 0.2% vehicle controls. Cytoplasmic eGFP Intensity was quantified and depicted as mean eGFP ± SEM (*n *= 3). Values were min–max normalized to subtract background signal, min = eGFP intensity at time point 0 h & max = maximum eGFP intensity at 24 h for CDDO-Me

To validate the application of the reporter hiPSCs for high content imaging (HCI), undifferentiated SBAD2-HMOX1-eGFP cells were exposed to DEM or CDDO-Me and imaged for 24 h (Fig. 5a). Confocal imaging revealed that eGFP expression was localised in the cytoplasm and increased over 24 h (Fig. 5b). Images were quantified and single-cell data were visualised as mean eGFP intensity over time of exposure (Fig. 5c, d), and a dose-dependent increase was observed in eGFP intensity after around 4 h of CDDO-Me and DEM exposure. EGFP intensity displayed a peak at around 16 h of 177.83 nM CDDO-Me exposure, after which the signal intensity stabilized (Fig. 5d), consistent with the Western blot data (Fig. 2b). CDDO-Me concentrations higher than 316.23 nM displayed a toxic phenotype indicated by lowered nuclear counts, increased fraction of propidium iodide (PI) positive cells and decreased 72 h ATP levels (Supplementary Fig. 8a, b, Supplementary Fig. 9). Despite inducing toxicity, 563.34 nM and 1000 nM CDDO-Me did not affect cytoplasm integrity and eGFP expression was still induced over the 24 h treatment period, at a lowered intensity (Fig. 5d). In contrast, cells exposed to toxic DEM concentrations (177.83–1000 µM) did not retain cytoplasm integrity and instead displayed a gradual decrease in eGFP intensity (Fig. 5d). This was coupled with a reduced nuclear count, increased PI expression and loss of 72 h ATP (Supplementary Fig. 8c/d, Supplementary Fig. 9). In conclusion, the HCI screen demonstrated the sensitivity of the SBAD2-HMOX1-eGFP reporter system in its ability to depict concentration specific responses as well as identifying time dependent dynamics of the oxidative stress response-related HMOX1 induction in hiPSCs.

### Multi-lineage high content imaging of SBAD2-HMOX1-eGFP

**Fig. 6:**
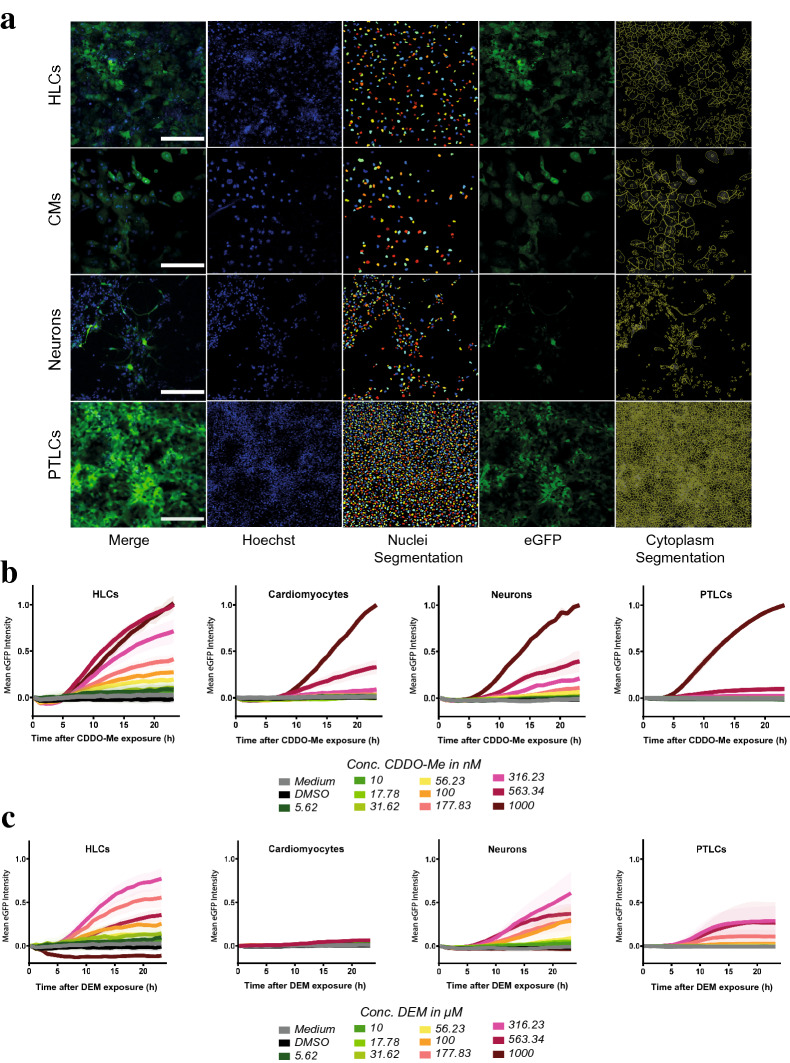
High content confocal imaging of differentiated SBAD2-HMOX1-eGFP hiPSC-derived reporter cells. **a** Example shown of hepatocyte-like cells (HLCs), cardiomyocyte-like cells (CMs), neuron-like cells (neurons) and proximal tubule-like cells (PTLCs) after 24 h CDDO-Me exposure. For all lineages HMOX1-eGFP channel was used for cytoplasmic segmentation (green), whilst nuclear segmentation was performed on the Hoechst channel (blue). Object segmentation parameters captured varying cellular morphologies. Scale bar is 200 µm. **b** Differentiated SBAD2-HMOX1-eGFP hiPSC-derived reporter cells exposed to ten concentrations of CDDO-Me for 24 h alongside medium and DMSO 0.2% vehicle controls. HLCs (*n *= 5), CMs (*n *= 2), neurons (*n *= 3), PTLCs (*n *= 3). Cytoplasmic HMOX1-eGFP intensity was quantified and depicted as mean eGFP ± SEM. Values were min–max normalized to subtract background signal, min = eGFP intensity at time point 0 h & max = maximum eGFP intensity at 24 h for CDDO-Me. **c)** Differentiated SBAD2-HMOX1-eGFP hiPSC-derived reporter cells exposed to ten concentrations of DEM for 24 h alongside medium and DMSO 0.2% vehicle controls. HLCs (*n *= 5), CMs (*n *= 3), neurons (*n *= 3), PTLCs (*n *= 2). Min–max normalized eGFP intensity is depicted as mean eGFP ± SEM

To uncover cell-specific oxidative stress responses and to validate the reporter functionality in multiple lineages, we repeated the HCI experiment (Fig. 5a) in the previously described differentiated SBAD2-HMOX1-eGFP cell cultures (Fig. 3a, b). 24 h live cell HCI of cells exposed to CDDO-Me and DEM revealed cytoplasmic eGFP induction in all four lineages (Fig. 6a, Supplementary Fig. 7). Due to differences in cytoplasmic morphology, lineage-specific image segmentation parameters were used for single-cell HMOX1 quantification (Fig. 6a). The quantified single-cell data were visualised as mean eGFP intensity over time of exposure (Fig. 6b, c). Since different cell lineages could not be imaged at the same time, raw intensity values were normalized per cell type and, therefore, only allowing comparison across lineages at a qualitative level and not at the absolute reporter intensity level.

hiPSCs reduced their sensitivity to CDDO-Me- and DEM-induced toxicity evenly across lineages after differentiation. Across lineages only two treatment conditions resulted in an absent (1000 µM DEM) or decreased (563.34 µM DEM) eGFP induction, coupled with a direct cytotoxic phenotype (Fig. 6c, Supplementary Fig. 8, Supplementary Fig. 9).

Of the four differentiated lineages, HLCs showed the most consistent HMOX1-reporter response. For both CDDO-Me and DEM we observed a similar, dose-dependent regulation of reporter activity (Fig. 6b, c). 1000 and 563.34 nM CDDO-Me expressed equal eGFP intensities without affecting cell viability, suggesting that maximal reporter activation had been reached (Fig. 6b, Supplementary Fig. 8a-b). Neuron-like cells also displayed clear dose-dependent increase of eGFP expression in response to both oxidative stress-inducing compounds (Fig. 6b, c). The eGFP expression extended through to the axoplasm and notably only a partial eGFP response was exhibited within the cell population (Fig. 6a, Supplementary Fig. 7). CMs showed a reduced and delayed oxidative stress response, with eGFP induction starting around 7.5 and 10 h, for CDDO-Me and DEM, respectively (Fig. 6b, c). Over 24 h, only the three highest CDDO-Me concentrations (316.23, 563.34 and 1000 nM) induced a steep and dose-dependent increase in eGFP intensity (Fig. 6b), while in the case of DEM exposure low and slowly increasing eGFP signals were observed (Fig. 6c, Supplementary Fig. 7). After 72 h a clear dose-dependent decrease in ATP was seen for DEM in CMs, suggesting the response peaked somewhere between 24 and 72 h (Supplementary Fig. 9).

PTLCs also only responded to the three highest CDDO-Me concentrations, with the 1000 nM response curve showing a large separation from 316.23 and 563.34 nM, which instead stabilized around 12 h (Fig. 6b). DEM exposure induced a more uniform dose response separation and eGFP stabilisation was again observed (Fig. 6c). Normally, cytotoxic phenotypes were induced systematically throughout the well, PTLCs instead displayed an uneven distribution of living and dead cells after DEM exposure. Imaged areas contained both PI positive/eGFP negative cells alongside PI negative/eGFP positive cells (Supplementary Fig. 7, Supplementary Fig. 8d). This phenomenon led to a high variability in mean eGFP intensities (Fig. 6c).

To provide a quantitative comparison of the HCI findings, we extrapolated the data and determined the point of departures (PoDs) for eGFP induction for both CDDO-Me and DEM in the different lineages (Supplementary Fig. 10). This allowed us to pinpoint the exact concentrations at which CDDO-Me and DEM activated the oxidative stress response, giving insights into lineage-specific sensitivities for chemical-induced oxidative stress (Table 1).

**Table 1:** Point of departure concentrations (PoD) determined for CDDO-Me and DEM in five SBAD2-HMOX1-eGFP lineages using confocal imaging data at time point 24 h

	CDDO-Me (nM)	DEM (µM)
hiPSC	91	44
HLC	137	104
CM	236	259
Neurons	160	39
PTLC	345	61

For CDDO-Me, PoDs ranged from 91 to 345 nM where hiPSCs displayed the lowest PoD followed by HLCs, neuron-like cells, CMs and finally PTLCs. This trend was not conserved across stressors as DEM determined PoDs sensitivities ranging from 39 to 259 µM for neuron-like cells, hiPSCs, PTLCs, HLCs and CMs. In conclusion, HCI of SBAD2-HMOX1-eGFP line proved to be successful in displaying lineage and compound-specific sensitivities. The sensitive HMOX1 biomarker enabled the detection of the oxidative stress response for compounds and concentrations which may otherwise be overlooked when using more conventional detections methods such as ROS dyes (Supplementary Fig. 11). Coupled with PoD modelling the application of the hiPSC reporter system can provide a sensitive and efficient tool for oxidative stress response prediction in vitro.

## Discussion

There is an increasing need for reliable cellular test systems in the field of toxicology. CRISPR/Cas9-assisted genome editing offers a powerful tool to generate improved in vitro models through tagging of endogenous stress response genes in cell lines relevant for chemical safety assessment. Here, we have presented the generation and extensive characterization of a fluorescent HMOX1 reporter hiPSC line, which was used to visualise and accurately quantify the chemically induced oxidative stress response in a variety of stem cell derived lineages.

To establish the reporter cell line, a protein-based CRISPR/Cas9 system was used, known to be more effective, immediate and transient, thus less harmful to the cells than vector-based approaches. Due to a relatively short exposure to the delivered CRISPR/Cas9-components, there is a lower risk for off-target events (Kim et al. 2014b; Liang et al. 2015; Lin et al. 2014). Gene targeting resulting in a mixed cell population, required a refined selection process to enrich for the successful HDR-edited cells and to create homogeneous cell lines through subcloning, achieved using a PiggyBac flanked selection cassette in the presented study. The targeting strategy was designed based on general considerations and according to the nature and molecular structure of HMOX1. N-terminal tagging of HMOX1 was excluded due to the risk of disturbing the endogenous regulatory mechanisms and the normal expression of the targeted gene (Majewski and Ott 2002). In general, fusion tagging is the most preferred strategy to generate endogenously tagged reporter cells, allowing for monitoring of the subcellular localization and expression-dynamics of the encoded fusion protein. However, HMOX1 is known to be inserted into the ER-membrane by a special mechanism through the TRC40/Get pathway, that is unique for the tail-anchored proteins (Borgese et al. 2003; Borgese and Fasana 2011; Shao and Hegde 2011; Wang et al. 2011). In case of C-terminal fusion-tagging the tail position of the transmembrane domain may be changed and shifted toward the middle part of the protein. As a result, the HMOX1-eGFP fusion protein would no longer be recognized by the regulators of the special membrane insertion pathway, which may affect the normal localization and function of the protein. Accordingly, C-terminal 2A-tagging was chosen as the most suitable strategy in case of this gene. Notable consequence of using 2A-tagging strategy is that eGFP is more stable in the cells than HMOX1 (half-lives ~ 26 h versus 15–21 h, respectively) (Corish and Tyler-Smith 1999; Dennery 2001; Srivastava et al. 1993), allowing for extended detection of the signal, thereby providing a powerful reporter system.

We reported on a large-scale study to fully validate the specificity and sensitivity of the new hiPSC HMOX1-eGFP reporter system using a HCI platform. We optimised our imaging pipelines to quantify eGFP in lineage-specific morphologies and confirmed reporter functionality in hiPSCs and four hiPSC-derived lineages. During HCI we observed variations in basal HMOX1 expression amongst lineages, further confirmed at the transcriptomic level. Although it led to a loss of comparable HMOX1-eGFP values, optimisation of optical configuration for different lineages is essential to ensure the detection of weak signal while preventing image oversaturation. HCI also revealed that PI was a poor indicator of cell death for neuron-like cells with high variability and low induction over 24 h. PI staining has been applied for neuronal cell counts through accumulation in the Nissl Bodies which could explain the high fraction of PI positive cells at 0 h (de Calignon et al. 2009; Niu et al. 2015).

Our HCI platform was able to detect the dynamic activation of HMOX1 amongst different lineage types, and we reported differences relating to time of induction and to concentration sensitivities. The application of reporters for in vitro chemical safety assessment should be paired with the ability to accurately identify concentrations that elicit the activation of the stress response, as this can predict an increased risk for adversity (Wink et al. 2017). Despite limitations in eGFP stabilisation, PoD modelling successfully predicted compound-specific sensitivities for all lineages. This gives us confidence that the SBAD2-HMOX1-eGFP reporter line can accurately predict the HMOX1 specific oxidative stress response in vitro*.*

Distinct oxidative stress response dynamics were displayed by cardiomyocyte-like cells in regard to their delayed eGFP induction. Transcriptomics analysis revealed that CMs uniquely expressed high basal levels of KEAP1 (Fig. 6c). Since DEM and CDDO-Me perturb the binding of KEAP1-CUL3 complex to NRF2 by targeting the KEAP1 sensor Cys151, an abundance of KEAP1 molecules in CMs might require more time and electrophilic binding before NRF2 can activate the oxidative stress response (Saito et al. 2016). Also, we observed that CMs expressed higher levels of *GCLC* than the other lineages, suggesting improved capacity to synthesize GSH, thereby protecting the CMs against oxidative stress.

The HLC lineage displayed by far the most adaptive oxidative stress response during HCI towards both CDDO-Me and DEM, responding already at lower concentrations and inducing few toxic phenotypes (nuclear decrease and increased PI positive fraction), independent of the stressor. This was in line with the lineage-specific DEG expression profile indicating functions relating to detoxification and metabolism, thereby priming HLCs with a reducing environment enabling an effective adaptive response towards oxidative stress (Gu and Manautou 2012). As such, hepatocytes might be more flexible and adaptive in dealing with oxidative stress conditions. Given the capacity of hepatocytes to bioactivate xenobiotics to reactive metabolites, such a versatile response to such harmful conditions might be an essential characteristic to provide an efficient adaptive response.

The strength of the hiPSC reporter system lies in the ability to efficiently screen multiple lineages originating from the same genetic background, thus removing donor-specific variations as found in multi-lineage screens. Lineage maturity of hiPSC-derived cells, however, remains a concern for the toxicology field but advances in 3D and co-culture differentiation protocols will allow for further representation of the in vivo situation (Kumar et al. 2020). TempO-Seq analysis identified *HMOX1* as a sensitive and highly inducible biomarker, which plays an important role in a stress response conserved across lineages and throughout different in vitro or in vivo models (Limonciel et al. 2018; McMahon et al. 2018). This proof-of-concept study allowed us to confirm the application of the SBAD2-HMOX1-eGFP reporter cells for in vitro safety prediction using HCI and demonstrated that HMOX1 is a powerful biomarker for oxidative stress induction. We foresee that this will be a powerful tool for toxicology and risk assessment, being accurate and high-throughput in its application.

## Supplementary Information

Below is the link to the electronic supplementary material.

Supplementary file1 (DOCX 57 KB)

Supplementary file2 (PDF 31247 KB)

Supplementary file3 (XLSX 80 KB)

## Data Availability

The datasets generated during and/or analysed during the current study are available from the corresponding author on reasonable request.
