# Supplementary material for: Fluorescent tagging of endogenous Heme oxygenase-1 in human induced pluripotent stem cells for high content imaging of oxidative stress in various differentiated lineages
Source: Arch Toxicol. 2021 Sep 4;95(10):3285–302. doi: 10.1007/s00204-021-03127-8 (PMC8448683; doi:10.1007/s00204-021-03127-8)
Supplement: Supplementary file 1 — Supplementary file1 (DOCX 57 KB) [file 204_2021_3127_MOESM1_ESM.docx]

#### SUPPLEMENTARY MATERIALS and METHODS

**gRNA design, synthesis and testing.** Guide RNA sequences were designed using the <http://CRISPR.mit.edu/> CRISPR design tool (Supplementary Table 1). To synthesize the gRNAs, template oligonucleotides were annealed and *in vitro* transcription (IVT) was performed using MEGAshortscript™ T7 Transcription Kit (Thermo Fisher Scientific), according to the manufacturer’s protocol. The gRNAs were tested for their function by *in vitro* DNA cleavage assay using GeneArt™ Platinum™ Cas9 nuclease (Thermo Fisher Scientific) and a DNA cleavage template amplified from SBAD2 genomic DNA, containing the target sites in the *HMOX1* gene (gene ID: 3162). To test gRNA activities in a genomic environment 8x10^5^ SBAD2 hiPSCs were nucleofected with CRISPR/Cas9 ribonucleoprotein (RNP) complexes composed of GeneArt™ Platinum™ Cas9 and guide RNAs (15 µg). Two days later the cells were harvested, and genomic DNA was analysed by T7E1-assay to estimate the efficiency of the targeted DNA-cleavage.

**Donor vector design and construction.** The donor vector contained the eGFP coding sequence at the 3’-end of the *HMOX1* left homology arm (LHA) and a selection cassette in the right homology arm (RHA) consisting of an EF1α-promoter-driven puromycin resistance gene. PiggyBac inverted terminal repeats (ITRs) were included, flanking the selection cassette and providing a tool for excision of the resistance gene. 1kb-long homology arms were inserted into the vector, designed from the GRCh38 reference genome and synthesized as GeneArt™ Strings DNA Fragments (Thermo Fisher Scientific), to ensure efficient homologous recombination. A single PAM point-mutation was introduced to destroy Cas9 recognition and gRNA binding and prevent the cleavage of the donor vector by the CRISPR/Cas9 system.

**PCRs, qPCRs, RT-qPCRs.** Overlapping junction PCRs and reactions for checking cassette excision were performed using locus-specific genomic primers that bind outside of the homology arms, in combination with vector-specific primers (Supplementary Figure S2, Supplementary Table 3). The fragments were amplified by Phusion Hot Start II High-Fidelity DNA Polymerase (Thermo Fisher Scientific). For qPCR-based copy number measurements, genomic DNA was subjected to PCR using eGFP TaqMan probe (Mr00660654_cn, Thermo Fisher Scientific) and TaqMan™ Universal PCR Master Mix (Thermo Fisher Scientific). RnaseP was used for normalization as this gene is known to have stable 2 copies per diploid genome (TaqMan® Copy Number Reference Assay, 4403326, Thermo Fisher Scientific). For RT-qPCR measurements total RNA was isolated using RNeasy Plus Mini Kit (QIAGEN) and 500 ng of RNA was transcribed with Maxima First Strand cDNA Synthesis Kit for RT-qPCR with dsDNase (Thermo Fisher Scientific), according to the manufacturer’s protocol. The amplification reactions were carried out in a total volume of 15 μl using SYBR Green JumpStart Taq ReadyMix. Oligonucleotide primers detailed in Supplementary Table 3 were designed using the Primer3 software ([Rozen and Skaletsky 1999](#_ENREF_4)) and verified by BLASTn (http://blast.ncbi.nlm.nih.gov/Blast.cgi). Human GAPDH was used as a reference gene. qPCR and RT-qPCR reactions were run on the Rotor-Gene Q 5plex Platform (QIAGEN) and data were analysed using the REST software (2009; version 2.0.13).

**Off-target analysis.** The most likely off-target sites were predicted by the CCTop CRISPR design tool and the corresponding genomic regions were PCR-amplified using Phusion Hot Start II High-Fidelity DNA Polymerase (Finnzymes). PCR products were puriﬁed with the Monarch® PCR & DNA Cleanup Kit (New England Biolabs) and sequenced directly using an ABI Prism 3130xl Genetic Analyzer and BigDye Terminator Cycle Sequencing v3.1 Kit (Applied Biosystems).

**Southern blot.** 5 μg gDNA was digested overnight with EcoNI (NEB) and separated on agarose gel, then transferred onto Hybond N+ nylon membrane (Amersham). The DIG-labeled DNA probe was prepared by random primed labeling of the eGFP sequence (720 bp). Probe labeling, hybridization and detection were performed using DIG-High Prime DNA Labeling and Detection Starter Kit II (Roche), following instructions of the manufacturer.

**Karyotyping.** SBAD2-HMOX1-eGFP reporter hiPSCs were treated with Demecolcine solution (10 μg/ml in HBSS) and processed with standard methods. Giemsa-banded karyotype analysis was performed on a minimum of 20 metaphase cells and the chromosomes were classiﬁed according to the International System for Human Cytogenetic Nomenclature (ISCN).

**Pluripotency tests, *in*** ***vitro* spontaneous differentiation.** Cell clumps were cultured in suspension for ﬁve days in mTeSR-1. The formed embryoid bodies (EBs) were plated on 0.1% gelatin (Merck) coated surface in differentiation medium (DMEM, 20% FBS, 1% MEM Non-Essential Amino Acid Solution (100x), 0.1 mM β-mercaptoethanol, 1% Pen/Strep). On day 14 of differentiation cells were collected for TaqMan hPSC Scorecard Analysis or ﬁxed with 4% PFA and evaluated for the 3 germ layer markers by immunocytochemistry as well.

**Immunocytochemistry (ICC).** Cells were fixed in 4% paraformaldehyde (PFA) for 20 minutes at room temperature, washed twice with PBS, and permeabilized with 0.2% Triton X-100 in PBS for 20 minutes. Cells were blocked with 3% bovine serum albumin (BSA) in the presence of 0.2% Triton X-100 in PBS for 60 minutes at room temperature. The respective primary antibodies were applied overnight at 4°C (Supplementary Table 4). After that, cells were incubated for 60 minutes at room temperature with the appropriate secondary antibodies (Supplementary Table 4). Cell nuclei were visualized using ProLong™ Diamond Antifade Mountant with 4′,6-diamidino-2-phenylindole (Thermo Fisher Scientific). Cells were analysed under a fluorescence microscope equipped with a 3D imaging module (Axio Imager system with ApoTome; Zeiss) controlled using AxioVision 4.8.1 software (Zeiss).

**Directed differentiation of hiPSCs into hepatocyte-like cells.** For hepatocyte like cell differentiation, we used the protocol recently described in ([Boon et al. 2020](#_ENREF_1)). Differentiation of SBAD2-HMOX1-eGFP iPSCs was performed on Matrigel-coated 96-well Screenstar microplates (Greiner Bio-One) for 28 days in liver differentiation medium (LDM) (consisting of 60% DMEM low glucose; 40% MCDB-201; 1x-Penicillin-Streptomycin; (10,000 U/mL)); 0.25x LA-BSA (100x); 0.25x ITS-A (100x); 100 nM, L-ascorbic Acid; 1 μM dexamethasone; 50 μM, 2-mercaptoethanol (50 mM)) supplemented with the following cytokines: day 0 to 2: Activin (50 ng/mL) + Wnt3a (50 ng/mL); day 2-4 Activin (50 ng/mL); day 4-8 BMP4 (50 ng/mL); day 8-12 aFGF (20 ng/mL); and day 12-end of the culture HGF (20ng/mL) (all cytokines were from Peprotech). From day 0-12, 0.67% DMSO (Sigma-Aldrich) was added. From day 12-14, MEM Non-Essential Amino Acids Solution (100X) and MEM Amino Acids Solution (50X) (Thermo Fisher Scientific) (16 mL/100 mL LDM) medium was added to the culture medium, which was then made PH neutral through the addition of NaOH. From day 14-end of the culture, glycine was further supplemented at 20 g/L (Sigma-Aldrich).

**Directed differentiation of hiPSCs into cardiomyocytes-like cells** Differentiation of hiPSCs into cardiomyocytes was performed in cardiomyocyte differentiation medium (CDM) as previously described ([van den Berg et al. 2016](#_ENREF_6)). iPSCs were passaged as aggregates in mTeSR-1 (Stem Cell Technologies) onto Matrigel-coated 6-well culture plates. Once a confluency of 40-50% was reached, mTeSR-1 was replaced by CDM (1:1 ratio mixture of IMDM and F12 nutrient mixture (Ham) with glutaMAX, supplemented with 5% PFHM-II, 0.25% w/v BSA (Sigma-Aldrich), 1x Chemically Defined Lipid Concentrate, Insulin-Transferrin-Selenium-X, 450 µM α-MTG (Sigma-Aldrich), 0.05 mg/mL L-Ascorbic acid 2-phosphate (Sigma-Aldrich), 2 mM Glutamax supplement, 25 U/mL penicillin and 25 µg/mL streptomycin) supplemented with 20 ng/mL ActivinA (Peprotech), 20 ng/mL BMP4 (Peprotech) and 1.5 µM CHIR99021 (Axon Medchem). After monolayer formation at day 3 medium was replaced by CDM supplemented with 5 µM XAV939 (Tocris). From this point, the medium was changed every 3 days with fresh non-supplemented CDM. To enable live-cell imaging, cells were dissociated at day 15 with 1x TryplE Select and 200,000 cells/cm^2^ were seeded in a Matrigel-coated 96-well Screenstar microplate (Greiner Bio-One) in CDM supplemented with 1x RevitaCell. Cells were cultured until day 21 and subsequently exposed to various oxidative stress inducers.

**Directed differentiation of hiPSCs into neurons and glial-like cells.** For neural differentiation, a previously established protocol was used which is based on the dual inhibition of SMAD signalling ([Chambers et al. 2009](#_ENREF_2); [Shi et al. 2012](#_ENREF_5)). When the iPSC culture reached appropriate confluency the culturing medium was changed to Neuronal Induction Medium to initiate the differentiation toward the neuronal lineage (1:1 mixture of DMEM/F12 and neurobasal medium, N-2 supplement, B-27 supplement, nonessential amino acids (NEAA, Merck), 2 mM L-glutamine, 50 U/ml penicillin/streptomycin, 5 μg/ml insulin (Merck), supplemented with 5 ng/ml basic fibroblast growth factor (bFGF), 0.2 μM LDN193189 (Selleckchem) and 10 μM SB431542 [Merck]). During the induction, neuroepithelial cell sheets with rosette-like structures were formed. After two weeks of induction, the cells were plated onto poly-L-ornithine/laminin-coated surface (POL/L, Merck) and the neural progenitor cells (NPCs) were further cultured in Neural Maintenance Medium (1:1 mixture of DMEM/F12 and neurobasal medium, N-2 supplement, B-27 supplement, NEAA, 2 mM L-glutamine, 50 U/ml penicillin/streptomycin), supplemented with 10 ng/ml epidermal growth factor (Thermo Fisher Scientific) and 10 ng/ml bFGF. To obtain neuronal cultures containing cortical neurons and glial cells for imaging, the NPCs were dissociated with Accutase then plated on POL/L-coated 96-well Screenstar microplates (Greiner Bio-One) at a density of 45,000 cells/cm^2^ and cultured for a further 14 days. On day 15 medium was changed to Neural Differentiation Medium (1:1 mixture of DMEM/F12 and neurobasal medium, N-2 supplement, B-27 supplement minus antioxidants, NEAA, 2 mM L-glutamine, 50 U/ml penicillin/streptomycin) until day 21 of terminal differentiation (TD21) after which the cells were exposed to various concentrations of CDDO-Me (Selleckchem) and DEM (Merck) then analysed as described below.

**Directed differentiation of hiPSCs into proximal tubular-like cells**

Differentiation of iPSC into proximal tubular-like cells (PTLCs) was done as previously described by [Chandrasekaran et al. (2021)](#_ENREF_3). Cells were passaged into single cells with accutase and centrifuged at 300 g for 5 min. Cells were then resuspended in differentiation medium 1 (1:1 mixture of DMEM/F12 (Thermo Fisher Scientific), 2 mM Glutamax, and ITS (5 µg/mL, 5 µg/mL, 5 ng/mL, Sigma-Aldrich), supplemented with 3 µM CHIR99021 (Sigma-Aldrich), 1 µM TTNPB (Sigma-Aldrich) and 10 µM Rock inhibitor (abcam) and plated out at 35,000 cells per cm^2^ on Geltrex (reduced growth factor, Thermo Fisher Scientific) coated 96-well plates. After 42h (day 2) medium was replaced with differentiation medium 1 supplemented with 1 µM TTNPB. After 72h (day 3) medium was replaced with proximal tubular medium 2 (1:1 mixture of DMEM/F12 (Thermo Fisher Scientific), 2 mM Glutamax, ITS (5 µg/mL, 5 µg/mL, 5 ng/mL, Sigma-Aldrich), 10 ng/ml EGF (Sigma-Aldrich) and 36 ng/ml hydrocortisone (Sigma-Aldrich) supplemented with 10 ng/ml FGF9 (Thermo Fisher Scientific). On day 6, FGF9 was removed and cells were fed with fresh proximal tubular medium 2. Cells were then fed every 2 to 3 days with proximal tubular medium 2 until day 14.

**HepG2 and PHH culture.** HepG2 human liver carcinoma cells were purchased from ATCC (clone HB8065). Cells were cultured in DMEM high glucose (Gibco, Thermo Fisher Scientific) and supplemented with 10% FBS (GE Healthcare), 25 U/ml penicillin and 25 μg/ml streptomycin (Thermo Fisher Scientific). Cells were passaged using trypsin-EDTA and 156250 cells/cm^2^ cells were seeded onto 96 well culture plates (Corning). Cryopreserved primary human hepatocytes consisting of a pool of 10 individual donors were purchased from BioIVT. Cells were thawed in OptiThaw media (Sekisui XenoTech) and resuspended in InVitroGRO CP hepatocyte plating media (BioIVT) supplemented with Torpedo antibiotic mix (BioIVT). Post thawing viability was determined using Trypan Blue and 218750 cells/cm^2^ were plated onto Collagen I coated 96well culture plates (BioCoat, Corning). Media was refreshed 6 h post-plating and switched to InVitroGRO HI hepatocyte maintenance media (BioIVT) supplemented with Torpedo antibiotic mix (BioIVT). Both HepG2 and PHH were cultured at 37°C in a humidified atmosphere containing 5% CO2.

**HepG2 and PHH compound exposure and transcriptomic analysis.** Compound exposure was performed 48 h post-seeding for HepG2s and 24 h post-seeding for PHHs in their respective maintenance media. Cells were treated for 24 h with CDDO-Me (3 to 3300 nM) and DEM (10 to 3300 µM). Compounds were prepared following the procedures described for SBAD2 HMOX1-eGFP reporter cells. All exposures were completed in triplicate on cells originating from three independent experiments. TempO-Seq transcriptomic samples were analysed following the same pipeline as described for SBAD2 HMOX1-eGFP reporter cells. HepG2 samples for 3300 µM DEM treatment had read counts lower than 100,000 and were excluded for further analysis. Due to an updated probe set not all NRF2 target genes (as defined by DoRothEA v.2) overlapped between the PHH, HepG2, and HLC data sets resulting in the exclusion of some genes.

**PoD determination**. Quantified mean eGFP intensity values at 24 h, from the HCI screen, were plotted along the log concentration for each lineage and stressor. Extrapolated point of departures (PoDs) were determined as the lowest concentration at which a significant (DMSO 0.2% + 4x the standard deviation) eGFP indication was observed.

**ATPlite viability assay**. Cell viability was assessed 72 h post-exposure on all 5 cell lineages using ATPlite 1 step Luminescence Assay (PerkinElmer) following manufacturer instructions. Briefly, the exposure medium was removed and replaced with 50 µL DMEM/F-12 without phenol red. 50 µL of the reconstituted ATPlite 1 step reagent was added to all wells and incubated on an orbital microplate shaker at 500 rpm for 2 minutes whilst shielded from light. Luminescence was measured using a FLUOstar OPTIMA FL Microplate Reader.

**ROS detection assay**. SBAD2 wild type hiPSCs were differentiated into cardiomyocytes, exposed to CDDO-Me (5.62 to 1000 nM) or DEM (5.62 to 1000 µM) and imaged over 24h. Tert-butylhydroperoxide (TBHP, Sigma-Aldrich) was added as a positive control at 50, 100 and 200 µM. For the detection of reactive oxygen species (ROS) 0.1 µM of dihydrorhodamine 123 dye (DHR123, Sigma-Aldrich) was added to the compound exposure media. Excitation by a 488 nm laser resulted in emission detection of the oxidized fluorescent R123, which localised in the mitochondria. Experiments were performed in triplicate, originating from three independent differentiations. The mean eGFP intensity was calculated over the three replicates and no additional image normalization was performed.

References

Boon R, Kumar M, Tricot T, Elia I, Ordovas L, Jacobs F, One J, De Smedt J, Eelen G, Bird M et al. 2020. Amino acid levels determine metabolism and CYP450 function of hepatocytes and hepatoma cell lines. *Nature Communications* **11**: 1393.

Chambers SM, Fasano CA, Papapetrou EP, Tomishima M, Sadelain M, Studer L. 2009. Highly efficient neural conversion of human ES and iPS cells by dual inhibition of SMAD signaling. *Nature biotechnology* **27**: 275-280.

Chandrasekaran V, Carta G, da Costa Pereira D, Gupta R, Murphy C, Feifel E, Kern G, Lechner J, Cavallo AL, Gupta S et al. 2021. Generation and characterization of iPSC-derived renal proximal tubule-like cells with extended stability. *Sci Rep* **11,** 11575 (2021). https://doi.org/10.1038/s41598-021-89550-4

Rozen S, Skaletsky H. 1999. Primer3 on the WWW for General Users and for Biologist Programmers. in *Bioinformatics Methods and Protocols* (eds. S Misener, SA Krawetz), pp. 365-386. Humana Press, Totowa, NJ.

Shi Y, Kirwan P, Livesey FJ. 2012. Directed differentiation of human pluripotent stem cells to cerebral cortex neurons and neural networks. *Nature protocols* **7**: 1836.

van den Berg CW, Elliott DA, Braam SR, Mummery CL, Davis RP. 2016. Differentiation of Human Pluripotent Stem Cells to Cardiomyocytes Under Defined Conditions. *Methods Mol Biol* **1353**: 163-180.
