# Supplementary material for: Fluorescent tagging of endogenous Heme oxygenase-1 in human induced pluripotent stem cells for high content imaging of oxidative stress in various differentiated lineages
Source: Arch Toxicol. 2021 Sep 4;95(10):3285–302. doi: 10.1007/s00204-021-03127-8 (PMC8448683; doi:10.1007/s00204-021-03127-8)
Supplement: Supplementary file 2 — Supplementary file2 (PDF 31247 KB) [file 204_2021_3127_MOESM2_ESM.pdf]

Supplementary Figures & Tables

Fluorescent tagging of endogenous Heme oxygenase-1 in human induced pluripotent stem cells for high content imaging of oxidative stress in various differentiated lineages

Kirsten E Snijders, Anita Fehér, Zsuzsanna Táncos, István Bock, Annamária Téglási, Linda van den Berk, Marije Niemeijer, Peter Bouwman, Sylvia E Le Dévédec, Martijn J Moné, Rob Van Rossom, Manoj Kumar, Anja Wilmes, Paul Jennings, Catherine M Verfaillie, Julianna Kobolák, Bas ter Braak, András Dinnyés and Bob van de Water

a

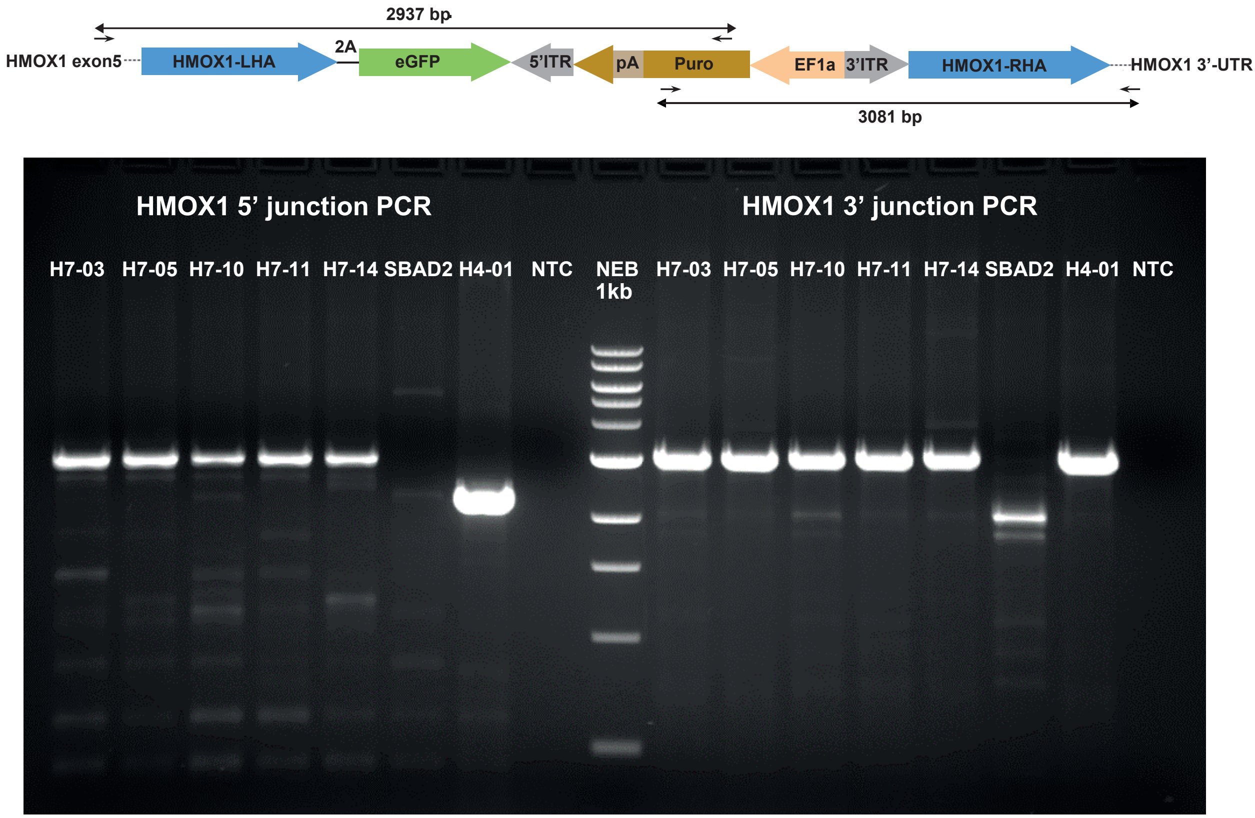

b

Samples	Relative copy number	Std. Error	Estimated copy number
NS13_11_Control	1	0,937 - 1,068	1
SBAD2_NF10 H7-03	0,96	0,856 - 1,071	1
SBAD2_NF10 H7-05	2,10	1,935 - 2,255	2
SBAD2_NF10 H7-10	1,18	1,064 - 1,312	1
SBAD2_NF10 H7-11	1,35	1,259 - 1,459	1
SBAD2_NF10 H7-14	1,93	1,813 - 2,092	2

Supplementary Fig. 1 Genetic screening and clone-characterization post knock-in.

a) Overlapping junction PCRs were performed using locus-specific primers that bind to genomic sequences outside of the homology arms, in combination with vector-specific primers for the puromycin resistance gene. Five clones tested positive in the screening that contained correctly integrated donor DNA at both ends. SBAD2: negative control, H4-01: HMOX1-targeted clone with incorrectly integrated donor DNA, NTC: no template control. **b)** Calculation of the genome integrated eGFP copy number in the candidate clones. Number of vector-insertions was evaluated by qPCR measurements (eGFP-specific TaqMan assay). Control: verified human iPSC line with one copy of genome integrated eGFP sequence.

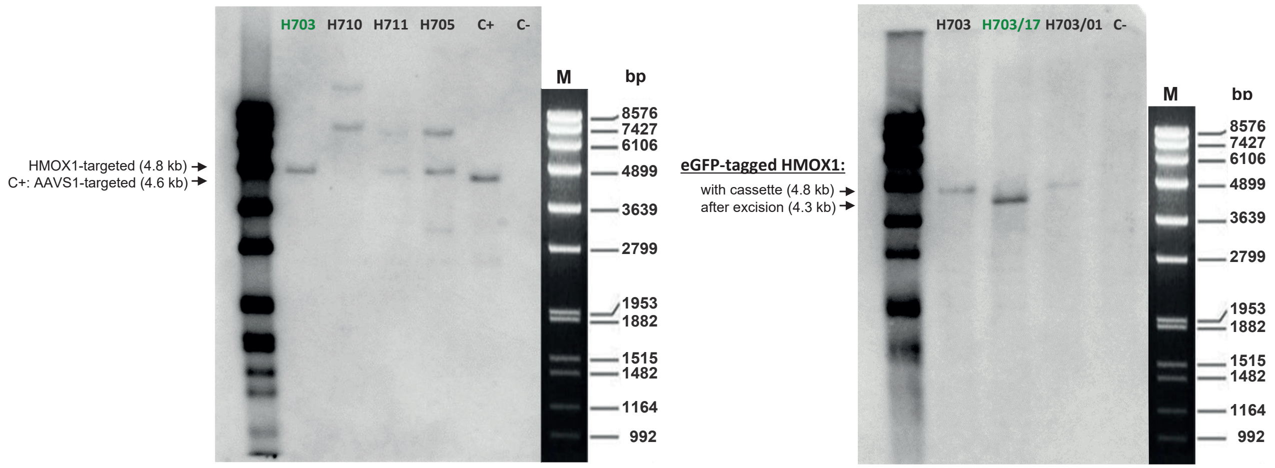

Supplementary Fig. 2 Southern blot analysis of candidate clones before and after cassette excision.

The results demonstrated a single-copy vector insertion in the targeted HMOX1 locus in the H7-03 cells (left panel) and confirmed the successful cassette removal in the H703/17 subclone (right panel). The samples were tested with eGFP-specific probe. C+: positive control hiPSC line containing genome integrated eGFP sequence in the AAVS1 locus, C-: negative control SBAD2 hiPSC line, M: DIG-labeled DNA Molecular Weight Marker VII (Roche).

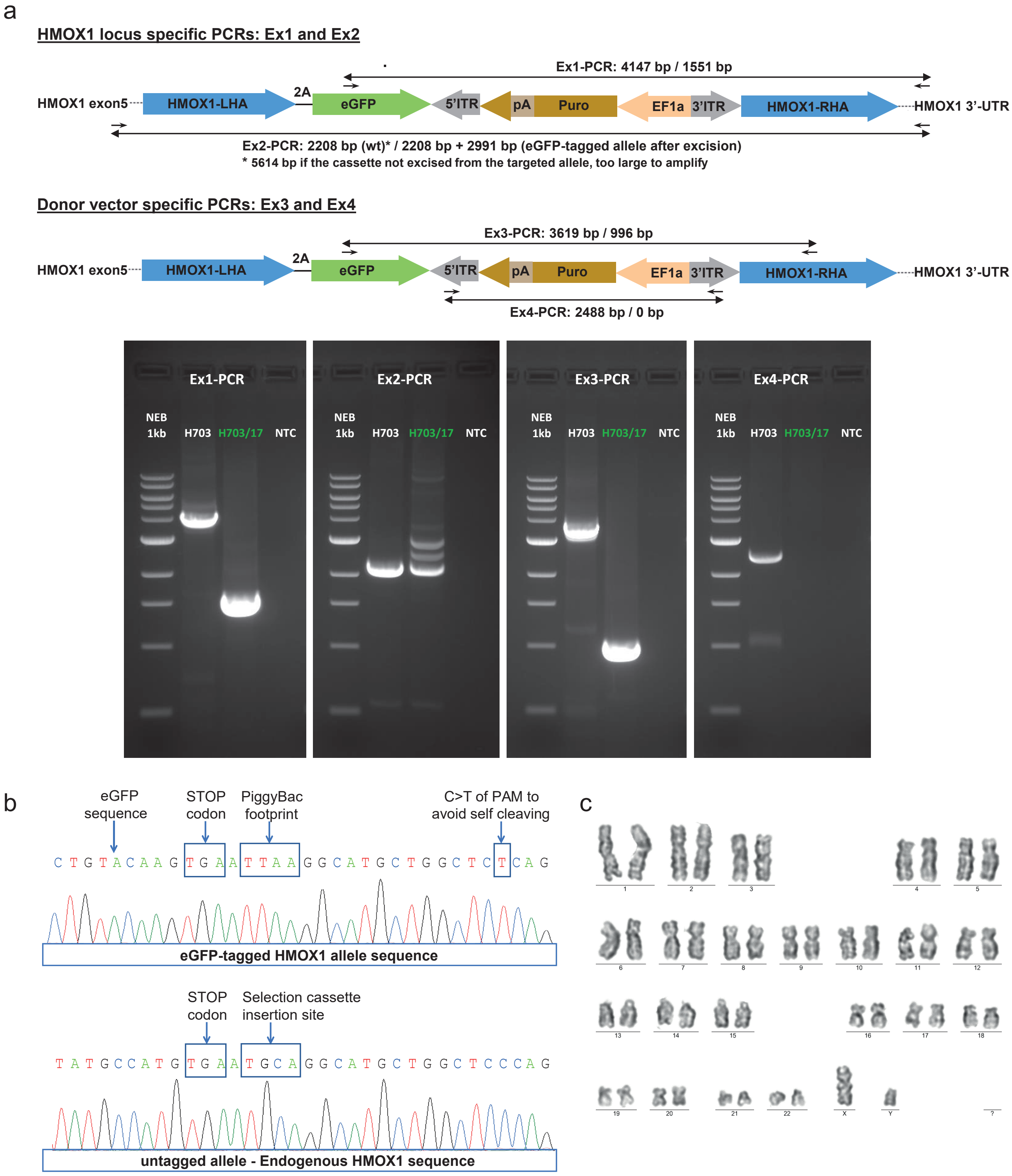

Supplementary Fig. 3 Genetic screening and clone-characterization post excision.

a) Removal of the selection cassette from the targeted HMOX1 locus was tested by PCRs using locus-specific genomic primers, that bind outside of the homology arms (Ex1-PCR, Ex2-PCR) and vector-specific primers, that bind to the eGFP- or piggyBac ITR-sequences (Ex1-PCR, Ex3-PCR, Ex4-PCR). **b)** Sanger-sequencing of the eGFP-tagged and untagged HMOX1 allele after successful cassette removal by the transposase. **c)** Karyogramm of H703/17 subclone showed normal 46 chromosomes (XY).

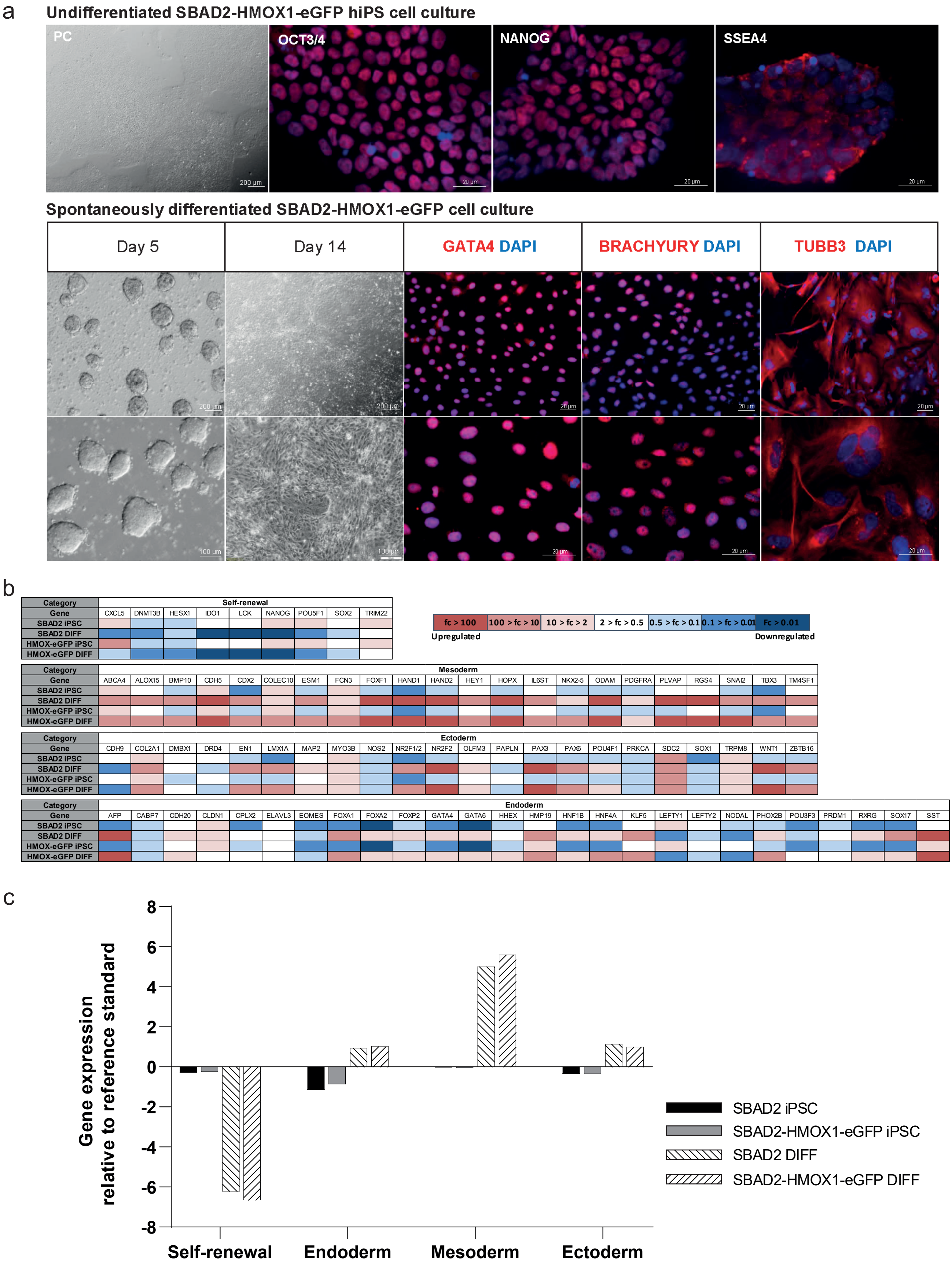
a
Undifferentiated SBAD2-HMOX1-eGFP hiPS cell culture
PC
OCT3/4
NANOG
SSEA4
200 µm
20 µm
Spontaneously differentiated SBAD2-HMOX1-eGFP cell culture
Day 5
Day 14
GATA4 DAPI
BRACHYURY DAPI
TUBB3 DAPI
100 µm
b
Category
Gene
Self-renewal
CXCL5 DNMT3B HESX1 IDO1 LCK NANOG POU5F1 SOX2 TRIM22
SBAD2 iPSC
SBAD2 DIFF
HMOX-eGFP iPSC
HMOX-eGFP DIFF
fc > 100
100 > fc > 10
10 > fc > 2
2 > fc > 0.5
0.5 > fc > 0.1
0.1 > fc > 0.01
Fc > 0.01
Upregulated
Downregulated
Mesoderm
ABCA4 ALOX15 BMP10 CDH5 CDX2 COLEC10 ESM1 FCN3 FOXF1 HAND1 HAND2 HEY1 HOPX IL6ST NKX2-5 ODAM PDGFRA PLVAP RGS4 SNAI2 TBX3 TM4SF1
Ectoderm
CDH9 COL2A1 DMBX1 DRD4 EN1 LMX1A MAP2 MYO3B NOS2 NR2F1/2 NR2F2 OLFM3 PAPLN PAX3 PAX6 POU4F1 PRKCA SDC2 SOX1 TRPM8 WNT1 ZBTB16
Endoderm
AFP CABP7 CDH20 CLDN1 CPLX2 ELAVL3 EOMES FOXA1 FOXA2 FOXP2 GATA4 GATA6 HHEX HMP19 HNF1B HNF4A KLF5 LEFTY1 LEFTY2 NODAL PHOX2B POU3F3 PRDM1 RXRG SOX17 SST
c
Gene expression
relative to reference standard
8
6
4
2
0
-2
-4
-6
-8
Self-renewal
Endoderm
Mesoderm
Ectoderm
SBAD2 iPSC
SBAD2-HMOX1-eGFP iPSC
SBAD2 DIFF
SBAD2-HMOX1-eGFP DIFF

Supplementary Fig. 4 Pluripotency analysis of SBAD2-HMOX1-eGFP cell line.

a) SBAD2-HMOX1-eGFP hiPSCs were spontaneously differentiated and analyzed by immunocytochemistry. Upper panels: Representative immunofluorescent micrographs of undifferentiated SBAD2-HMOX1-eGFP hiPSCs, that were positively stained for stem cell markers OCT3/4, NANOG and SSEA4 (in red), nucleus was labeled with DAPI (in blue). Lower panels: Spontaneously formed embryoid bodies (Day 5) and their further differentiation in adherent culture (Day 14). Trilineage differentiation potential was confirmed by immunostaining for endodermal (GATA4), mesodermal (BRACHYURY) and ectodermal (TUBB3) germ layers (in red), the nucleus was labeled with DAPI (in blue). **b)** Expression of self-renewal factors and germ layer-specific genes in undifferentiated and spontaneously differentiated SBAD2 and SBAD2-HMOX1-eGFP cells was assessed by TaqMan hPSC Scorecard Assay. **c)** Scorecard results depicted as relative to reference standard, expression was summarized for self-renewal factors and germ layer specific marker genes.

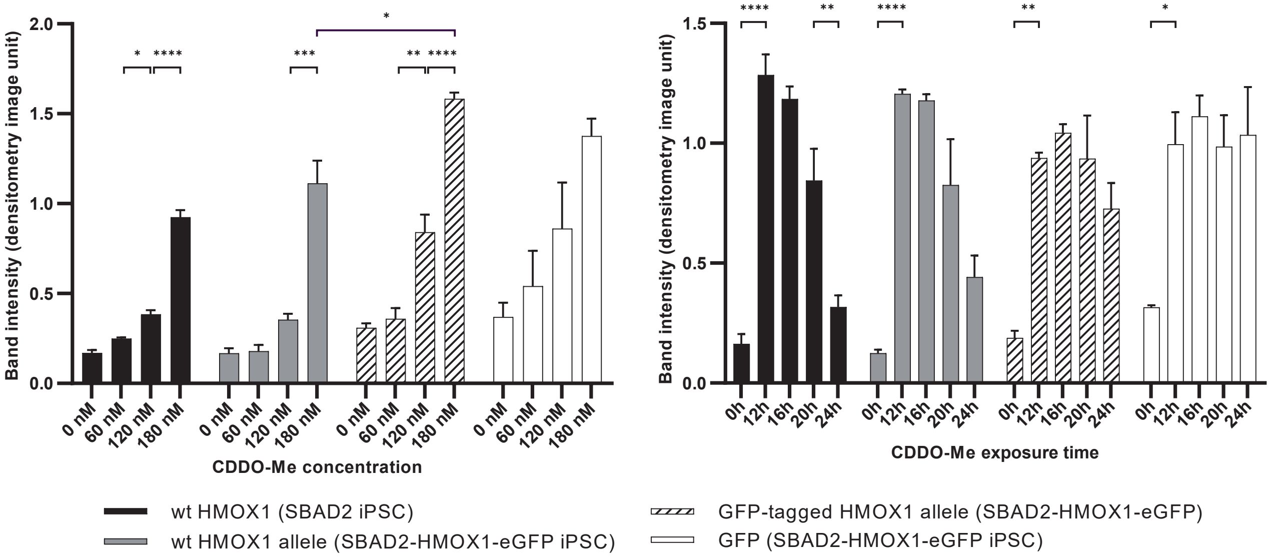

Supplementary Fig. 5 Quantification of the Western blot experiments performed on SBAD2 and SBAD2-HMOX1-eGFP hiPSCs to monitor the HMOX1-activation and eGFP protein expression dynamics under oxidative stress.

Results shown for 24 h exposure to varying concentrations of CDDO-Me as well as a kinetic study performed with 180 nM CDDO-Me sampled at different time points. All values were normalized to GAPDH and are presented as mean ± SEM (n=3). One-way ANOVA was used to assess the statistical significance of the differences (*$p<0.05$, **$p<0.01$, ***$p<0.001$, ****$p<0.0001$).

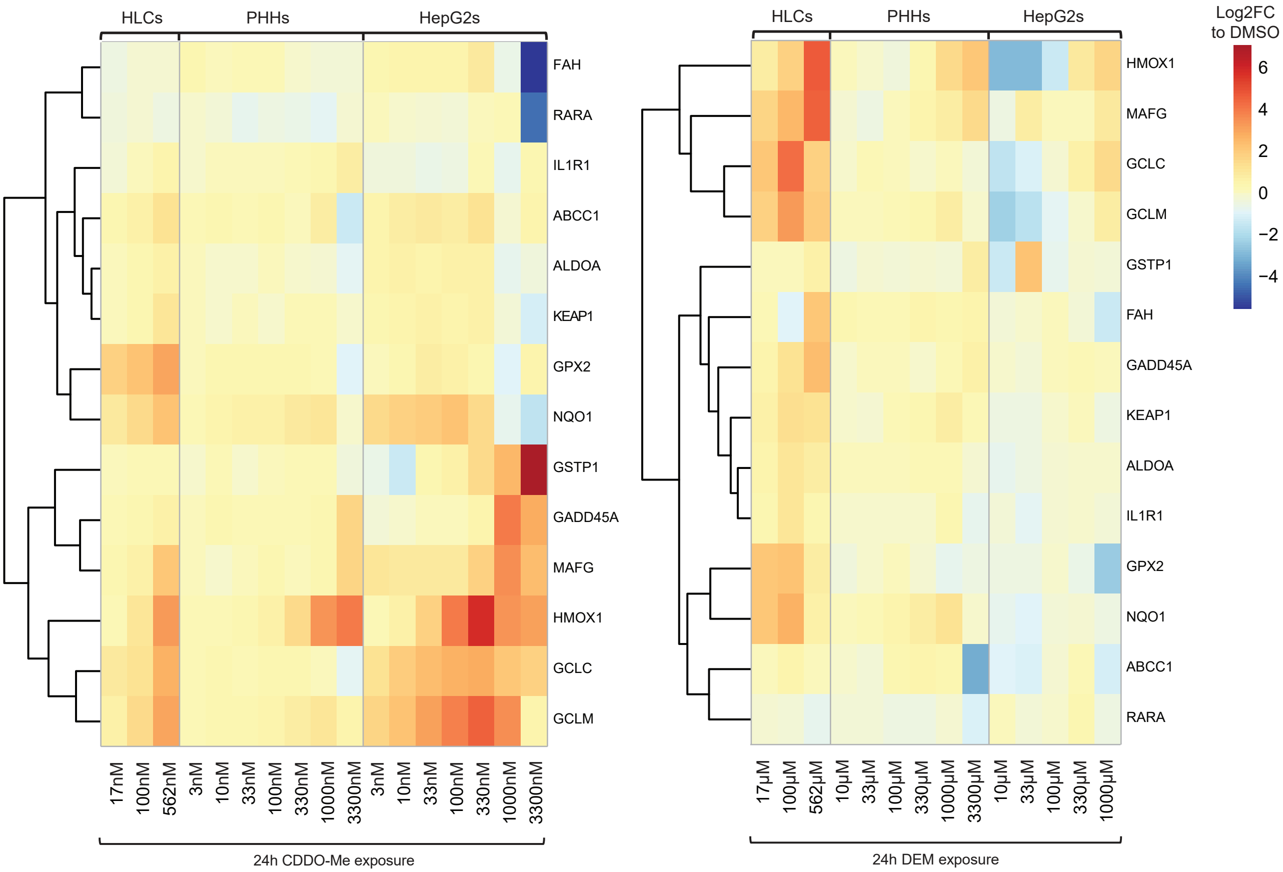

Supplementary Fig. 6 TempO-Seq analysis of oxidative stress response genes in three liver test systems.

TempO-Seq expression of NRF2 target genes in SBAD2-HMOX1-eGFP derived hepatocyte-like cells (HLCs), primary human hepatocytes (PHH) and HepG2 cells after 24 h CDDO-Me and DEM exposure where n=3. Displayed as log2 fold change compared to cell type specific DMSO 0.2% sample. Differentially expressed genes were selected based on the Dorothea downstream target selection tool with confidence A-C and were clustered according to the Euclidean distance metric.

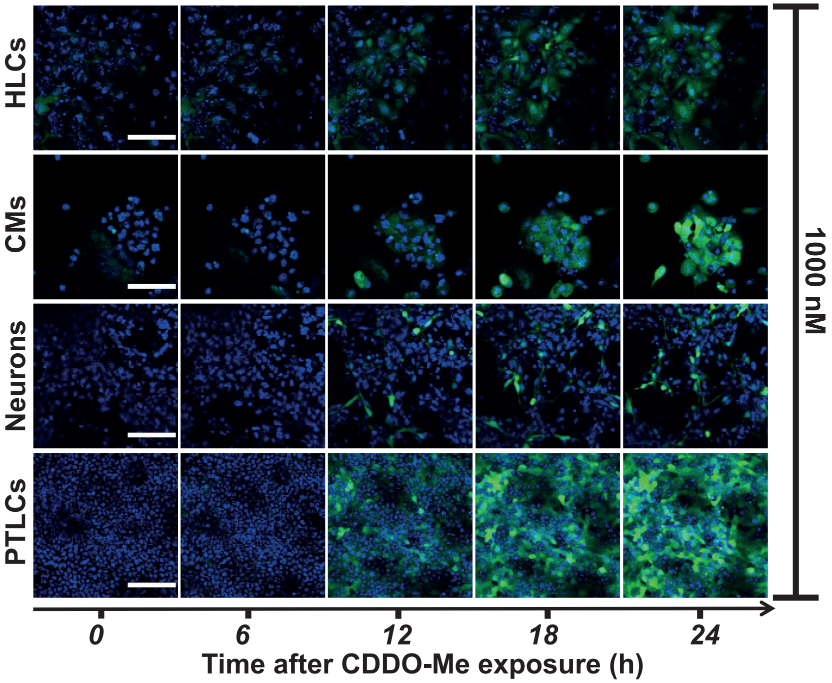

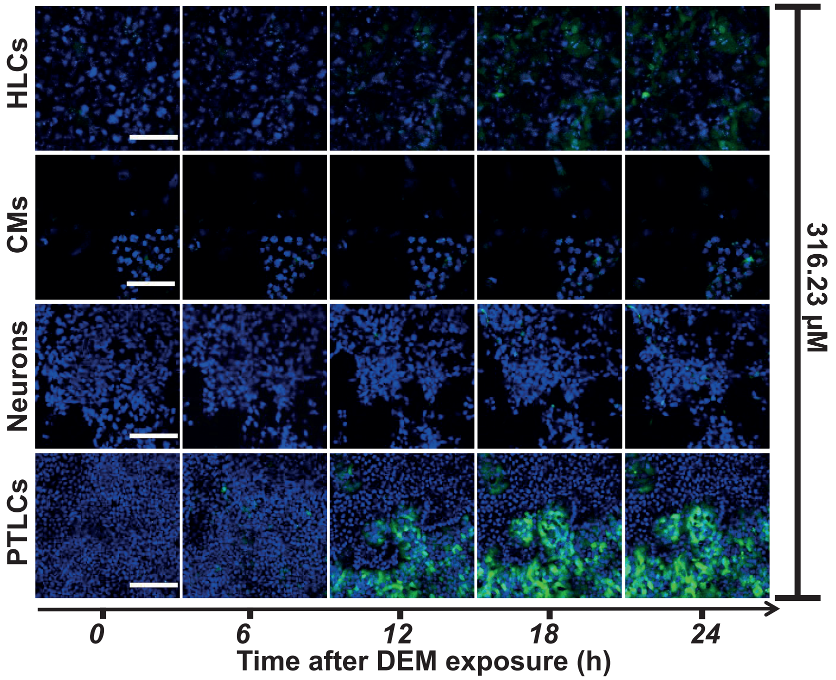

Supplementary Fig. 7 eGFP induction captured during high content imaging of oxidative stress induction.

Confocal images of differentiated SBAD2-HMOX1-eGFP reporter cells after exposure with 1000 nM CDDO-Me and 316.23 µM DEM over 24 h. Lineages include hepatocyte-like cells (HLCs), cardiomyocyte-like cells (CMs), neuron-like cells (neurons) and proximal tubule-like cells (PTLCs). Images represent the highest reached eGFP intensity per stressor. Hoechst stained nuclei visualized in blue and cytoplasmic eGFP visualized in green. Scale bar is 100 µm.

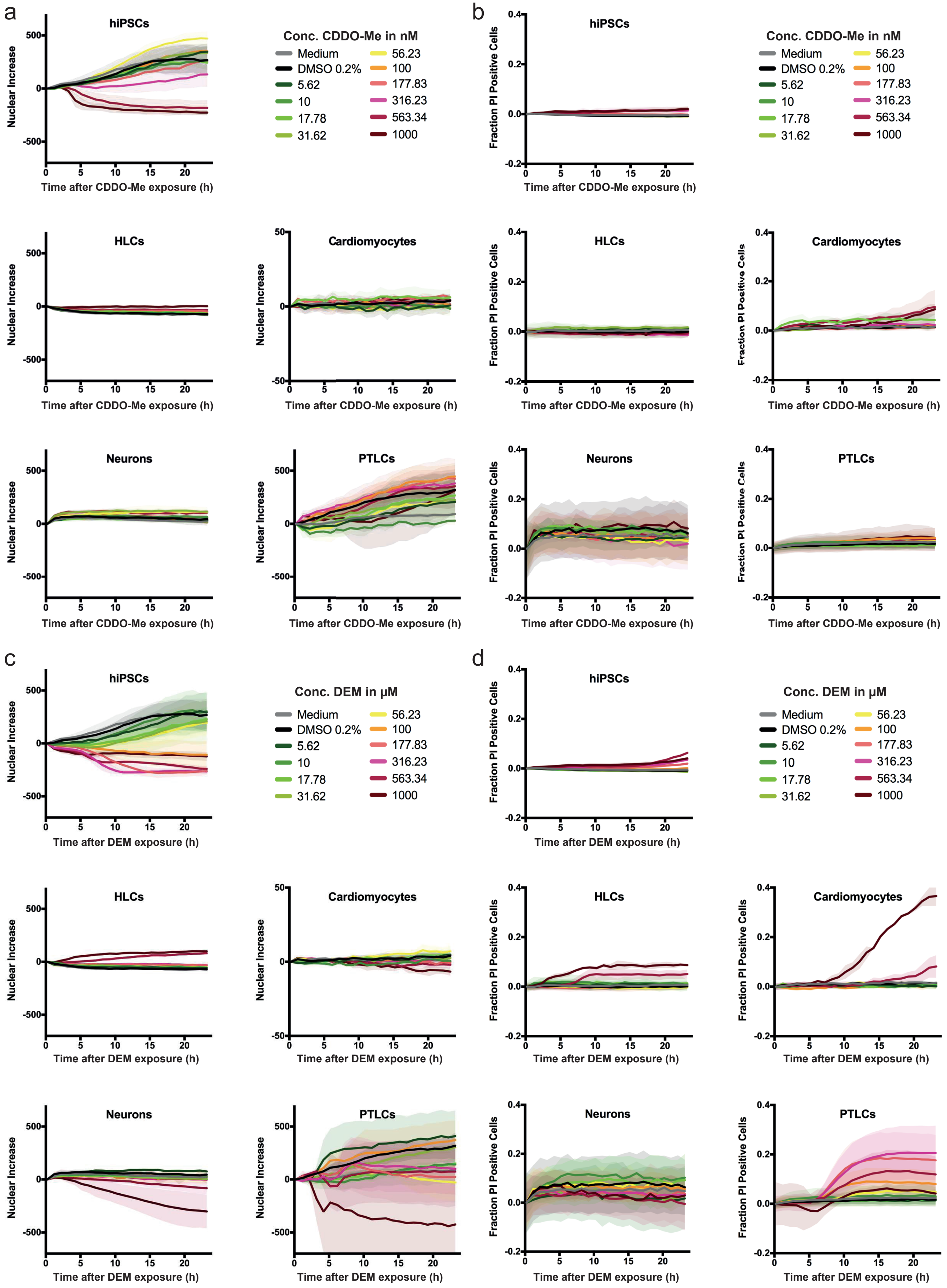
a
hiPSCs
Nuclear Increase
Time after CDDO-Me exposure (h)
Conc. CDDO-Me in nM
Medium
DMSO 0.2%
5.62
10
17.78
31.62
56.23
100
177.83
316.23
563.34
1000
HLCs
Cardiomyocytes
Neurons
PTLCs
b
Fraction PI Positive Cells
c
Time after DEM exposure (h)
Conc. DEM in μM
d

Supplementary Fig. 8 Quantitative assessment of cell viability using high content imaging.

SBAD2-HMOX1-eGFP reporter cells exposed to 10 concentrations of CDDO-Me for 24 h alongside medium and DMSO 0.2% vehicle controls [hiPSCs (n=3), hepatocyte-like cells (HLCs) (n=5), cardiomyocyte-like cells (cardiomyocytes) (n=2), neuron-like cells (neurons) (n=3), proximal tubule-like cells (PTLCs) (n=3)] and 10 concentrations of DEM for 24 h alongside medium and DMSO 0.2% vehicle controls [hiPSCs (n=3), HLCs (n=5), cardiomyocytes (n=3), neurons (n=3), PTLCs (n=2)]. **a/c)** Normalized nuclear count depicted as mean nuclear increase over time ± SEM. Values were normalised to cell count at time point 0 h. **b/d)** Fraction of PI positive cells over time depicted as mean ± SEM. Values were normalized to time point 0 h.

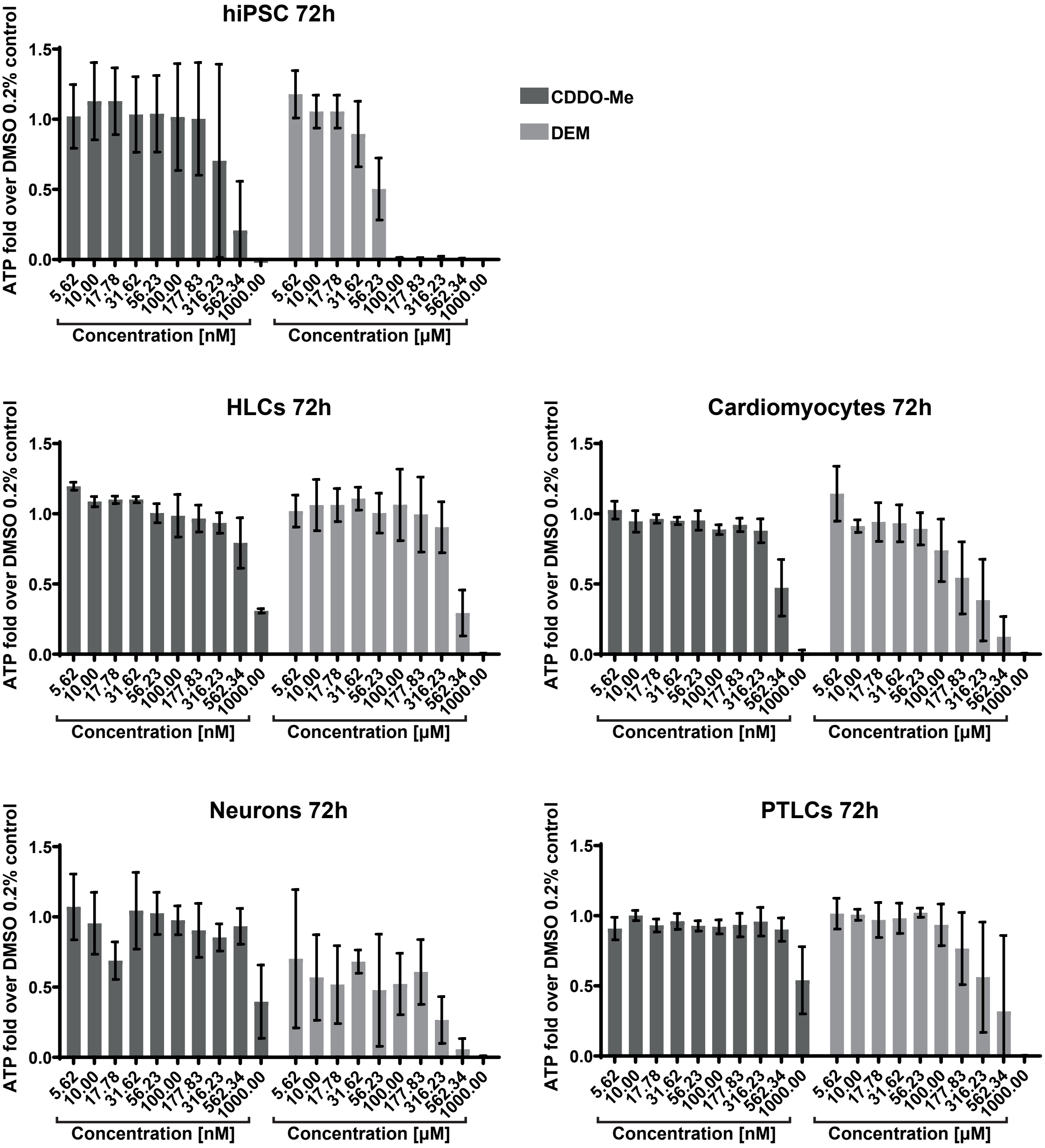

Supplementary Fig. 9 ATPlite assay on 5 lineages of SBAD2-HMOX1-eGFP reporter line measured 72 h post CDDO-e and DEM exposure.

Values are displayed as fold change over DMSO 0.2% control per lineage. For CDDO-Me: hiPSCs (n=3), HLCs (n=5), CM (n=2), neurons (n=3), PTLCs (n=3); and for DEM: hiPSCs (n=3), hepatocyte-like cells (HLCs) (n=5), cardiomyocyte-like cells (cardiomyocytes) (n=3), neuron-like cells (neurons) (n=3), proximal tubule-like cells (PTLCs) (n=2). Error bars indicate standard deviation.

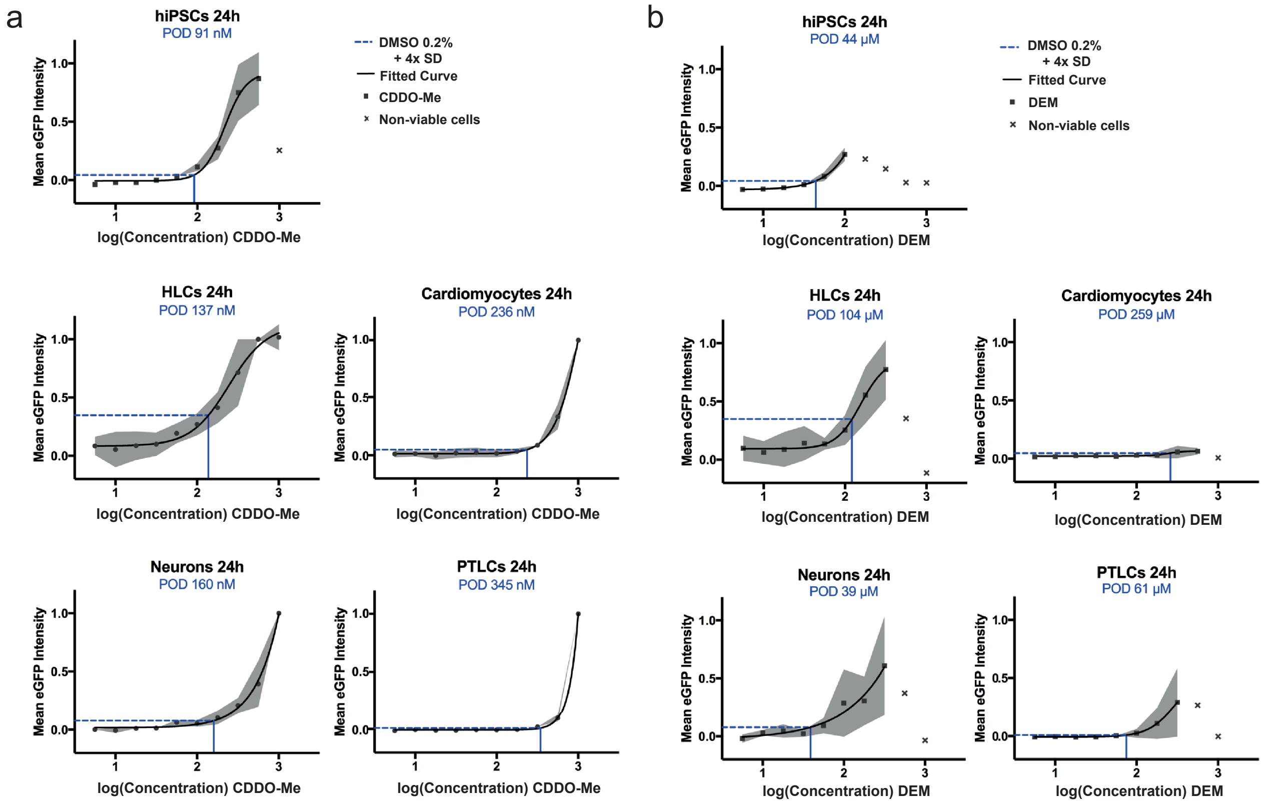

Supplementary Fig. 10 Point of departure analysis of CDDO-Me and DEM responses in different lineages.

SBAD2-HMOX1-eGFP hiPSC reporter cells exposed for 24 h with 10 concentrations of a) CDDO-Me and b) DEM. Lineages include hiPSCs, hepatocyte-like cells (HLCs), cardiomyocyte-like cells (cardiomyocytes), neuron-like cells (neurons) and proximal tubule-like cells (PTLCs). The presented values are the mean eGFP intensity ± SD after min-max normalization. Concentrations shown on a log scale and concentrations resulting in non-viable cells were excluded from the analysis. Point of departure (PoD) concentrations were determined at the intersection of the sigmoidal fitted curve and the control value (mean DMSO 0.2% + 4x SD).

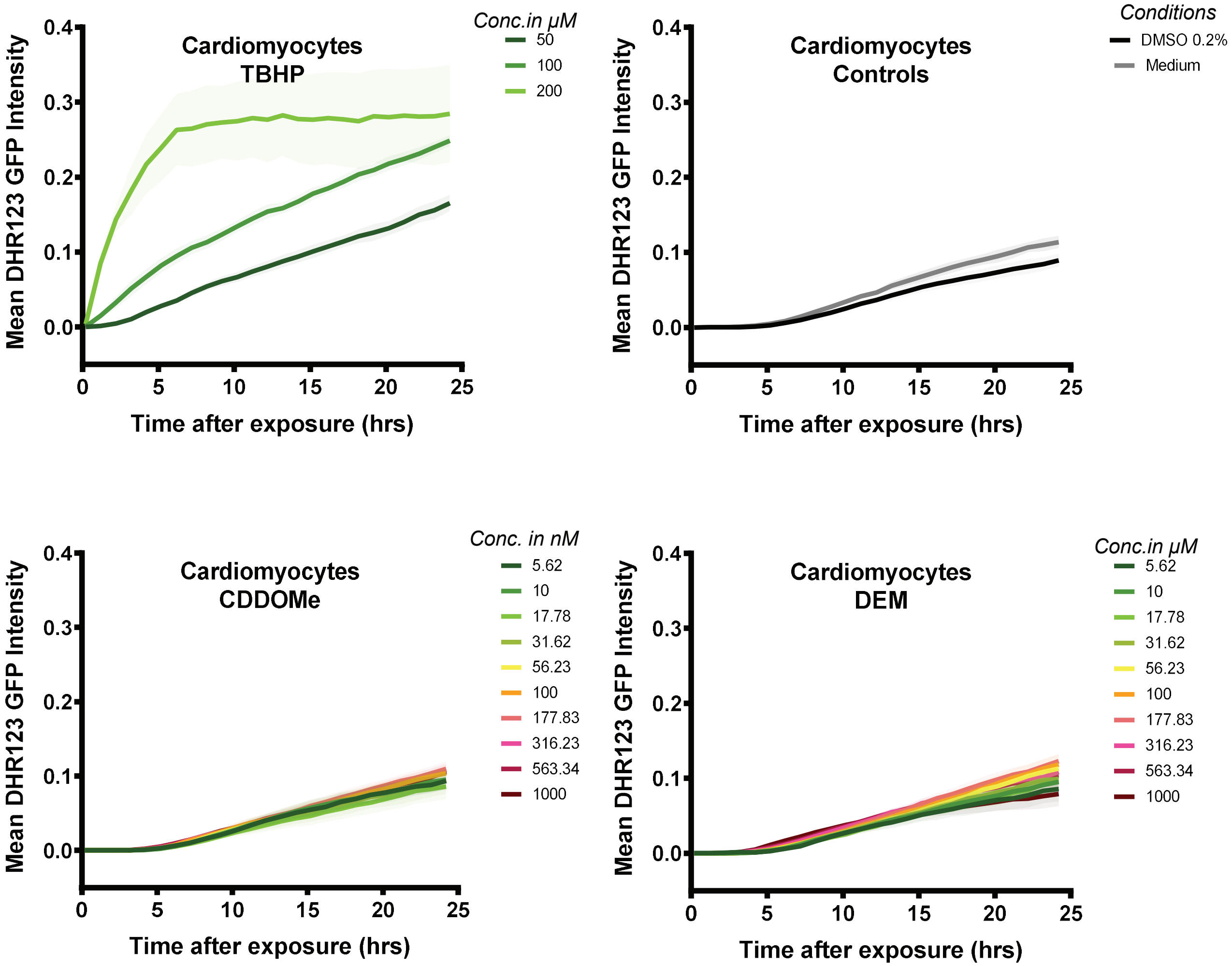

Supplementary Fig. 11 Quantification of oxidative stress induction in cardiomyocytes using DHR123 dye.

Cardiomyocytes derived from SBAD2 wild type cells exposed over 24 h to 10 concentrations of CDDO-Me and DEM, 3 concentrations of positive control TBHP, medium and DMSO 0.2% vehicle controls. Quantified single cell expression of oxidized R123 eGFP intensity is depicted as mean eGFP ± SEM (n=3).

Supplementary Table 1 Guide RNA sequences used in this study. The guide chosen to create the HMOX1-eGFP reporter iPSC line is *in italics*.

gRNA	Target sequence (5' – 3')	PAM	Strand	bp between the DNA cleavage site and HMOX1 STOP codon
HMOX1 gRNA1	GGGCCATGAACTTTGTCCGG	TGG	sense	38
HMOX1 gRNA2	CATGAACTTTGTCCGGTGGA	AGG	sense	42
HMOX1 gRNA3	*GGACAAAGTTCATGGCCCT*	*GGG*	*antisense*	*23*

Supplementary Table 2 Sequences of most likely off-target sites predicted for the HMOX1 gRNA (first raw). Mismatches (MM) are in red, gRNA core is in parentheses, PAM sequences are indicated with green boxes and the cleavage sites are shown by green arrows.

Coordinates	MM	target_seq	PAM	gene name	gene ID	seq_result
chr22:35393616-35393637	0	GGACAAA [GTTCATGGCCCT]	GGG	HMOX1	ENSG00000100292	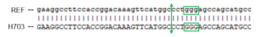
chr17:60073550-60073571	4	GTGTCAA [GTTCATGGCCCT]	CGG	HEATR6	ENSG00000068097	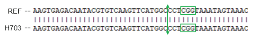
chr8:17632813-17632834	4	TGCCCAT [GTTCATGGCCCT]	GGG	PDGFRL	ENSG00000104213	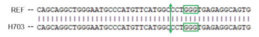
chr10:91698246-91698267	4	AGGAAAA [CTTCATGGCCCT]	GGG	GAPDHP28	ENSG00000213449	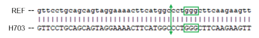
chr2:15502506-15502527	4	TGAGCAG [GTTCATGGCCCT]	AGG	NBAS	ENSG00000151779	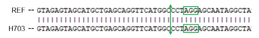
chr17:2526784-2526805	3	TGAGAAA [GTCCATGGCCCT]	GGG	METTL16	ENSG00000127804	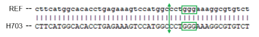

Supplementary Table 3 PCR primers used in the study.

Primer/PCR name	Forward sequence	Reverse sequence	Application
HMOX1-LHA-scr	5'-CCAGACCTAAGGCCCTGTTT-3'	5'-GATCCGGACCGCCACATC-3'	screen
HMOX1-RHA-scr	5'-TCGTAGAAGGGGAGGTTGC-3'	5'-AATTGCATCAAGCAGGGTTC-3'	screen
Ex1-PCR	5'-GAGCGCACCATCTTCTTCA-3'	5'-AATTGCATCAAGCAGGGTTC-3'	screen
Ex2-PCR	5'-CCAGACCTAAGGCCCTGTTT-3'	5'-AATTGCATCAAGCAGGGTTC-3'	screen
Ex3-PCR	5'-GAGCGCACCATCTTCTTCA-3'	5'-GCCACAGTGCCGTTAAACAC-3'	screen
Ex4-PCR	5'-GTCGCTGTGCATTTAGGACA-3'	5'-TTGACGCATGTGTTTTATCG-3'	screen
OFF1	5'-TTTCCAGCACTTGGTGACAG-3'	5'-CGTATGGCTGATGGGTAATG-3'	off-target
OFF2	5'-CCCACTTCTCCCAGAAACAA-3'	5'-CTAGAGCAGGGAGGGTGATG-3'	off-target
OFF3	5'-TCCAGAACCTTGGGACACTC-3'	5'-CCGCTGTCTGCATTTACTCA-3'	off-target
OFF4	5'-GCACACTCACTCACCCAAAG-3'	5'-GGTGATTTCGTCGTTGTGAA-3'	off-target
OFF5	5'-AACAAATGCAGAGGCCAGAC-3'	5'-AGCTGCCACAAACTTCAACC-3'	off-target
HMOX1	5'-CTGCCCTTCAGCATCCTCAGTT-3'	5'-GCTGCCACATTAGGGTGTCTT-3'	RT-qPCR
HMOX1-eGFP	5'-GGCCAGCAACAAAGTGCAAG -3'	5'-CCACGTCTCCAGCCTGCTTC-3'	RT-qPCR
GAPDH	5'-CTCTCTGCTCCTCCTGTTCGAC-3'	5'-TGAGCGATGTGGCTCGGCT-3'	RT-qPCR

Supplementary Table 4 List of antibodies used in this study for ICC.

	Antibody name	Host	Dilution	Cat #	Company
**Pluripotency**	anti-SSEA4	mouse	1:50	sc-59368	Santa Cruz Biotechnologies
	anti-OCT4	mouse	1:50	sc-5279	Santa Cruz Biotechnologies
	anti-NANOG	goat	1:50	AF1997	R&D
***In vitro* spontaneous differentiation**	anti-TUBB3	rabbit	1:2000	PRB-435P	Covance
	anti-BRACHYURY T	rabbit	1:50	sc-20109	Santa Cruz Biotechnologies
	anti-GATA4	mouse	1:50	sc-25310	Santa Cruz Biotechnologies
**Secondary antibodies**	Alexa Fluor 594 donkey anti-goat IgG	donkey	1:2000	A11058	Thermo Fisher Scientific
	Alexa Fluor 594 donkey anti-mouse IgG	donkey	1:2000	A21203	Thermo Fisher Scientific
	Alexa Fluor 594 donkey anti-rabbit IgG	donkey	1:2000	A21207	Thermo Fisher Scientific

Supplementary Table 5 EU-ToxRisk gene panel used for TempO-Seq analysis. (As separate supplementary)
